# Supplementary material for: Deciphering the Rules Underlying Xenogeneic Silencing and Counter-Silencing of Lsr2-like Proteins Using CgpS of Corynebacterium glutamicum as a Model
Source: mBio. 2020 Feb 4;11(1):e02273-19. doi: 10.1128/mBio.02273-19 (PMC7002338; doi:10.1128/mBio.02273-19)
Supplement: TEXT S1 [file mBio.02273-19-s0001.pdf]

SUPPLEMENTAL MATERIAL

Text S1: Supplementary information on methods used in this study

Growth conditions, monitoring of cell growth and fluorescence during microtiter cultivation and cultivation in the microfluidic chip device

Growth conditions

For all cultivations of *C. glutamicum* strains, brain heart infusion (BHI, Difco Laboratories, Detroit, MI, USA) complex medium was inoculated with a single colony from a fresh agar plate and incubated for 8 to 16 hours. All cultivation steps were performed at 30°C. For reporter-based assays, BHI pre-cultures were used to inoculate a second overnight pre-culture in CGXII minimal medium (1) supplemented with 25 µg/ml kanamycin and 100/111 mM glucose or 100 mM gluconate. Subsequently, the pre-culture was used to inoculate the main culture at a start OD_{600} of 1. Main cultures were cultivated in CGXII medium with 25 µg/ml kanamycin and 100/111 mM glucose, 100 mM gluconate or 100 mM fructose. *E. coli* DH5α and BL21 (DE3) were used for plasmid amplification and *cgpS* overexpression, respectively. Strains were cultivated in Lysogeny Broth (LB) media or on LB agar plates at 37°C. If needed, 50 µg/ml kanamycin was added. *Vibrio natriegens* Vmax^{TM} (Synthetic Genomics, San Diego, CA, USA) was used for GntR (Cg2783) protein production and cells were cultivated in BHIN complex medium (BHI + 15 g/l NaCl) supplemented with 50 µg/ml carbenicillin at 30°C.

Microtiter cultivation to monitor cell growth and fluorescence

Reporter-based analysis in microliter scale was performed in the BioLector® microcultivation system (m2p-labs, Aachen, Germany) (2). Therefore, 750 µl of the main culture (see Growth conditions) were cultivated in 48-well FlowerPlates (m2p-labs, Aachen, Germany) at 30°C and 1200 rpm. Biomass production was measured as backscattered light intensity of sent light with a wavelength of 620 nm (signal gain factor 20). Venus fluorescence was measured with an excitation wavelength of 508 nm and emission wavelength of 532 nm (signal gain factor 60). Samples were measured at 15 min intervals. Arbitrary units (a.u.) of specific fluorescence were calculated by dividing the Venus signal by the backscatter signal per time point (2). Obtained specific fluorescence values were background corrected by subtracting values of strains harboring the control plasmid pJC1-*venus*-term (no promoter in front of *venus*) (3) which were cultivated under comparable conditions. Shown fold changes were calculated based on the ratio of induced versus non-induced reporter outputs and correspond to reporter inducibility.

Cultivation in microfluidic chip device

The temporal dynamics of the genetic toggle switch were analyzed on single-cell level by cultivating *C. glutamicum* wild type cells harboring the plasmid-based construct in an in-house developed microfluidic platform (4, 5). The chip design and the experimental setup was performed as described before (6). Phase contrast as well as Venus and E2-Crimson fluorescence were imaged at 20 min intervals by fully motorized inverted Nikon Eclipse Ti microscope (Nikon GmbH, Düsseldorf, Germany) as described previously (4, 5, 7). The exposure times for phase contrast was 100 ms, for Venus 200 ms and for E2-crimson 300 ms. Cells were cultivated in the microfluidic chip system in CGXII medium supplemented

with 25 µg/ml kanamycin and either 100 mM gluconate or 111 mM glucose. Continuous medium supply with a flow rate of 200 nl/min and waste removal was achieved by a high-precision syringe pump system (neMESYS, Cetoni GmbH, Korbussen, Germany) using disposable syringes (Omnifix-F Tuberculin, 1 ml; B. Braun Melsungen AG, Melsungen, Germany). After 17 hours of cultivation, the carbon source supply was switched from gluconate to glucose or vice versa by changing the syringes and the connecting tubing to ensure an immediate medium change. The temperature was set to 30°C during the complete cultivation using an incubator system (PeCon GmbH, Erbach, Germany). Data analysis was performed using the image-processing package Fiji (8) which is based on ImageJ (9) on colony level. Obtained fluorescence data were background normalized and plotted with GraphPad prism 7.00 (GraphPad Software, La Jolla. CA. USA).

Construction of strain ∆phage::P$_{cgpS}$-*cgpS* and design of disruptive counter-silencing constructs

Construction of strain ∆phage::P$_{cgpS}$-*cgpS*

Re-integration of *cgpS* in the prophage-free strain ∆phage (MB001) (3) with its native promoter was performed with the pK19mobsacB-1199_1201-P$_{cgpS}$-*cgpS* integration plasmid. This plasmid contains the *cgpS* gene fused to its native promoter, which was flanked by 500 bp upstream and downstream regions of the integration site (intergenic region of cg1199-cg1201). Two step homologous recombination and selection was performed as described previously (10). Successful integration was verified by sequencing using the oligonucleotides cg1199_1201_seq_fw and cg1199_1201_seq_rv (Table S2G).

Design of disruptive counter-silencing constructs

Reporter studies were performed based on plasmid pJC1 (approximately 30 copies per cell) (11, 12). All counter-silencing constructs were based on the same design scheme. First, the native promoter region was amplified from *C. glutamicum* genomic DNA, fused to the gene *venus* via a consistent linker, which contains a ribosomal binding site, and inserted into the pJC1 plasmid using Gibson assembly (13). To ensure that all regulatory promoter elements are present, forward primers were designed so that the 5´-sequence end coincided with the upstream end of the CgpS binding peak (14). Reverse primers were chosen so that the promoter constructs contain the first 30 bp of the coding sequence. The resulting plasmids serve as template for the counter-silencer constructs. Overlap PCR was performed for the insertion of the GntR binding site (BS: TATGATAGTACCAAT) (15) at different positions. All constructed plasmids are listed in Table S2C and oligonucleotides are listed in Table S2D.

<u>Determination of transcriptional start sites (TSS)</u>

For the determination of the TSS, C. glutamicum wild type cells were cultivated in CGXII supplemented with 111 mM glucose starting at an OD_{600} of 1. After one-hour cultivation at 30°C, the SOS response was induced by adding 600 nM MMC leading to prophage induction (16). 50 ml cultures were harvested on ice after one, three and six hours of cultivation at 30°C, respectively. Pellets after centrifugation (5300 *g*, 4°C, 15 min) were snap-frozen in liquid nitrogen and stored at -80°C until use. Total RNA was prepared using the RNeasy Mini Kit (QIAGEN, Venlo, Netherlands) according to the manufacturer

protocol. Subsequently, all three RNA samples were pooled in approximately equal amounts. The determination of the TSS and data analysis was performed by Vertis Biotechnology AG (Vertis Biotechnology AG, Freising, Germany) using the Cappable-seq method developed by Ettwiller and Schildkraut (17). Obtained reads were mapped against the reference *C. glutamicum* genome BX927147 (18). Relative read score at a certain position and in a certain orientation (+ or - strand) (RRSio = (number of reads at position i in orientation o/total number of mapped reads)*1000000) were calculated by the company for a non-enriched control library (cutoff: 0) and for the enriched Cappable-seq library (cutoff: 5). The enrichment score (enrichment score = log2 (RRSio TSS / RRSio control) was calculated based on the RRSio of both libraries (cutoff: 3). Five bp of upstream and downstream region were used for clustering of TSS. Relevant TSS located in the promoter region (500 bp upstream of the start codon) and directed in gene orientation were assigned to relevant phage genes. Multiple TSS mapped to the same promoter were ranked depending on their enrichment scores (Table S1).

Analyses of AT-rich stretches in CgpS binding regions

Scanning of the *C. glutamicum* genome (BX927147 (18)) for AT-rich stretches was performed using a custom python script (submitted to GitHub: https://github.com/afilipch/afp/blob/master/genomic/get_at_stretches.py). AT-rich stretches were determined as the longest possible sequences with at least 70% adenosine/thymidine (AT)-content and a number of guanosines (G) and cytidines (C) below a particular threshold. The scan was performed multiple times with incrementing the limit for maximal allowed G/C interruptions inside AT-rich sequences. The results of

these multiple scans were then pulled together without further collapsing, meaning that the discovered AT-rich sequences with different G/C numbers may overlap each other. AT-rich regions were then grouped based on their lengths and G/C numbers (or number of AT steps). The grouping was done in a way to fulfil two requirements: the groups should be roughly equal and cover the whole dynamic range of the selected parameters. Subsequently, the fraction of AT-rich stretches overlapping (full overlap) with CgpS binding peaks were identified for each group (sequences were previously deposited in the GEO database (ncbi.nlm.nih.gov/geo) under accession number GSE80674).

Protein purification, surface plasmon resonance (SPR) spectroscopy and electrophoretic mobility shift assay (EMSA)

Protein purification

C-terminal Strep-tagged CgpS (Cg1966) was heterologously overproduced in *E. coli* BL21 (DE3) (19) harboring the plasmid pAN6-*cgpS*-Strep (14). Cell cultivation and protein purification was performed as described before by Pfeifer and colleagues (14) except for an increased amount of 15 mM d-desthiobiotin in the elution buffer. Purified Strep-tagged CgpS was used for surface plasmon resonance measurements.

GntR (Cg2783) with a 21 amino acids long N-terminal decahistidine tag (MGHHHHHHHHHHSSGHIEGRH) was heterologously overproduced in *Vibrio natriegens* Vmax™ (Synthetic Genomics, San Diego, CA, USA) harboring the plasmid pET16b-*gntR1* (15). Cells were grown in BHIN complex medium (BHI + 15 g/l NaCl) supplemented with 50 µg/ml carbenicillin and incubated at 30°C. Protein production was induced by the addition of 1 mM Isopropyl-β-D-1-thiogalactopyranoside (IPTG) at an OD_{600} of 0.3. After

four hours of subsequent cultivation, cells were harvested by centrifugation (5300 *g*, 4°C, 15 min). The pellet was resuspended in TNI20 buffer (20 mM Tris/HCl pH 7.9, 300 mM NaCl and 20 mM imidazole) and cells were disrupted by three passages through a French pressure cell (SLM Aminco, Spectronic Instruments, Rochester, NY, USA) at 172 mPa. To remove cell debris, cell lysate was centrifuged at 5300 *g*, 4°C for 15 min and subsequently ultracentrifuged for one hour (229000 *g*, 4°C). His-tagged GntR protein from the supernatant was purified with gravity-flow chromatography using Ni-NTA agarose columns (QIAGEN, Venlo, Netherlands). Columns were washed with TNI20 buffer followed by protein elution with TNI200 buffer (20 mM Tris/HCl pH 7.9, 300 mM NaCl and 200 mM imidazole). Protein fractions were pooled and buffer was exchanged against TG buffer (30 mM Tris/HCl pH 7.5, 10% (v/v) glycerol) using a PD-10 Desalting Column (GE Healthcare, Chicago, IL, USA). Subsequently, GntR was snap-frozen in liquid nitrogen and stored at -80°C before it was used for EMSAs.

Surface plasmon resonance (SPR) spectroscopy

Binding of Strep-tagged CgpS to native or synthetic target promoters was analyzed by SPR analysis in a Biacore T200 and a Biacore 3000 device (GE Healthcare, Freiburg, Germany) using carboxy-methyl dextran sensor chips pre-coated with streptavidin (XanTec SAD500L (XanTec Bioanalytics GmbH, Düsseldorf, Germany) and Sensor Chip SA (GE Healthcare, Freiburg, Germany). As first step, DNA-fragments covering the respective promoters or the promoter region of the gene cg3336 (negative control) were amplified by using biotinylated primer *via* two-step PCR as described in Table S2E. All experiments were carried out at a constant temperature of 25°C in HBS-EP+ buffer (10

mM HEPES pH 7.4, 150 mM NaCl, 3 mM EDTA, 0.05% (v/v) detergent P20). Before immobilization of the DNA fragments, the chips were equilibrated by three injections of 1 M NaCl/50 mM NaOH using a flow rate of 10 µl/min. Then, 10 nM of the respective double-stranded biotinylated DNA fragment was injected at a flow rate of 10 µl/min for a total contact time of 420 s. The chips were then washed by injecting 1 M NaCl/50 mM NaOH/50% (v/v) isopropanol. Approximately 300-550 RU (response units) of the relevant DNA fragment was bound per flow cell. Binding analysis of CgpS with the five DNA fragments P_{cg3336}, P_{cg1999}, P_{cg1999}_A-T/G-C, the native phage promoter P_{lys} or the corresponding counter-silencer construct P_{lys}_CS_0 were performed at a flow rate of 30 µl/min in HBS-EP+ buffer at 25°C. Various concentrations of CgpS (1 nM–5000 nM) dissolved in HBS-EP+ buffer, were passed over the flow cells for 180 s, and the complexes formed were allowed to dissociate for 420 s before the next cycle started. After each cycle, the surface was regenerated by injection of 2.5 M NaCl for 30 s, followed by 0.5% (w/v) SDS for 60 s, at a flow rate of 30 µl/min. All experiments were performed at 25°C. Sensorgrams were recorded using Biacore T200 Control Software 2.0 or Biacore 3000 Control Software 4.1.2, respectively, and analyzed with Biacore T200 Evaluation Software 2.0, BIAevaluation software 4.1.1 or TraceDrawer software 1.5 (Ridgeview Instruments, Uppsala, Schweden). The surface of flow cell 1 was not coated and used to obtain blank sensorgrams for subtraction of the bulk refractive index background. The referenced sensorgrams were normalized to a baseline of 0. Peaks in the sensorgrams at the beginning and the end of the injection are due to the run-time difference between the flow cells for each chip.

Electrophoretic mobility shift assay (EMSA)

Binding of GntR to the native P_{lys} promoter and the corresponding counter-silencer construct P_{lys}_CS_0 was analyzed by electrophoretic mobility shift assays (EMSAs). Primers and templates used for the PCR amplification of DNA fragments are listed in Table S2F. The PCR products were purified from an agarose gel with the PCR clean-up and gel extraction kit of Macherey Nagel (Düren, Germany). 14 nM DNA fragments covering the promoter regions (P_{lys}: 518 bp; P_{lys}_CS_0: 533 bp) were incubated for 15 min at room temperature with varying amounts (0, 28, 70, 140, 180 or 210 nM) of N-terminal tagged GntR (decahistidine tag) (15) in binding buffer (200 mM Tris/HCl pH 7.5, 200 mM KCl, 25 mM MgCl2, 25% (v/v) glycerol, 0.5 mM EDTA). If indicated, 100 mM gluconate or glucose was added to the binding buffer. All samples were loaded onto a native 10% polyacrylamide gel (loading dye: 0.01% (w/v) xylene cyanol dye, 0.01% (w/v) bromophenol blue dye, 20% (v/v) glycerol, 1xTBE (89 mM Tris base, 89 mM boric acid, 2 mM Na_2-EDTA)) and electrophoresis was performed at 170 V and room temperature with 1x TBE buffer. Gels were stained with SybrGreen I following the manufacturer instructions (Sigma-Aldrich, St. Louis, MO, USA).

REFERENCES

1. Keilhauer C, Eggeling L, Sahm H. 1993. Isoleucine synthesis in *Corynebacterium glutamicum*: molecular analysis of the *ilvB-ilvN-ilvC* operon. J Bacteriol 175:5595-5603.
2. Kensy F, Zang E, Faulhammer C, Tan RK, Büchs J. 2009. Validation of a high-throughput fermentation system based on online monitoring of biomass and fluorescence in continuously shaken microtiter plates. Microb Cell Fact 8:31.
3. Baumgart M, Unthan S, Rückert C, Sivalingam J, Grünberger A, Kalinowski J, Bott M, Noack S, Frunzke J. 2013. Construction of a prophage-free variant of *Corynebacterium glutamicum* ATCC 13032 for use as a platform strain for basic research and industrial biotechnology. Appl Environ Microbiol 79:6006-6015.
4. Grünberger A, Paczia N, Probst C, Schendzielorz G, Eggeling L, Noack S, Wiechert W, Kohlheyer D. 2012. A disposable picolitre bioreactor for cultivation and investigation of industrially relevant bacteria on the single cell level. Lab Chip 12:2060-2068.
5. Grünberger A, Probst C, Helfrich S, Nanda A, Stute B, Wiechert W, von Lieres E, Nöh K, Frunzke J, Kohlheyer D. 2015. Spatiotemporal microbial single-cell analysis using a high-throughput microfluidics cultivation platform. Cytometry A 87:1101-1115.
6. Grünberger A, Probst C, Heyer A, Wiechert W, Frunzke J, Kohlheyer D. 2013. Microfluidic picoliter bioreactor for microbial single-cell analysis: fabrication, system setup, and operation. J Vis Exp doi:10.3791/50560:e50560.

7. Helfrich S, Pfeifer E, Krämer C, Sachs CC, Wiechert W, Kohlheyer D, Nöh K, Frunzke J. 2015. Live cell imaging of SOS and prophage dynamics in isogenic bacterial populations. Mol Microbiol 98:636-650.

8. Schindelin J, Arganda-Carreras I, Frise E, Kaynig V, Longair M, Pietzsch T, Preibisch S, Rueden C, Saalfeld S, Schmid B, Tinevez JY, White DJ, Hartenstein V, Eliceiri K, Tomancak P, Cardona A. 2012. Fiji: an open-source platform for biological-image analysis. Nat methods 9:676-682.

9. Rueden CT, Schindelin J, Hiner MC, DeZonia BE, Walter AE, Arena ET, Eliceiri KW. 2017. ImageJ2: ImageJ for the next generation of scientific image data. BMC Bioinformatics 18:529.

10. Niebisch A, Bott M. 2001. Molecular analysis of the cytochrome bc_1-aa_3 branch of the *Corynebacterium glutamicum* respiratory chain containing an unusual diheme cytochrome c_1. Arch Microbiol 175:282-294.

11. Cremer J, Eggeling L, Sahm H. 1990. Cloning the *dapA dapB* cluster of the lysine-secreting bacterium *Corynebacterium glutamicum*. Mol Gen Genet 220:478-480.

12. Eggeling L, Bott M. 2008. The genus *Corynebacterium*, p 355-375. *In* Goldman E, Green LH (ed), Practical Handbook of Microbiology, 2nd ed. CRC Press, Boca Raton, FL, USA.

13. Gibson DG, Young L, Chuang RY, Venter JC, Hutchison CA, 3rd, Smith HO. 2009. Enzymatic assembly of DNA molecules up to several hundred kilobases. Nat Methods 6:343-345.

14. Pfeifer E, Hünnefeld M, Popa O, Polen T, Kohlheyer D, Baumgart M, Frunzke J. 2016. Silencing of cryptic prophages in *Corynebacterium glutamicum*. Nucleic Acids Res 44:10117-10131.

15. Frunzke J, Engels V, Hasenbein S, Gätgens C, Bott M. 2008. Co-ordinated regulation of gluconate catabolism and glucose uptake in *Corynebacterium glutamicum* by two functionally equivalent transcriptional regulators, GntR1 and GntR2. Mol Microbiol 67:305-322.

16. Nanda AM, Heyer A, Krämer C, Grünberger A, Kohlheyer D, Frunzke J. 2014. Analysis of SOS-induced spontaneous prophage induction in *Corynebacterium glutamicum* at the single-cell level. J Bacteriol 196:180-188.

17. Ettwiller L, Buswell J, Yigit E, Schildkraut I. 2016. A novel enrichment strategy reveals unprecedented number of novel transcription start sites at single base resolution in a model prokaryote and the gut microbiome. BMC Genomics 17:199.

18. Kalinowski J, Bathe B, Bartels D, Bischoff N, Bott M, Burkovski A, Dusch N, Eggeling L, Eikmanns BJ, Gaigalat L, Goesmann A, Hartmann M, Huthmacher K, Krämer R, Linke B, McHardy AC, Meyer F, Möckel B, Pfefferle W, Pühler A, Rey DA, Rückert C, Rupp O, Sahm H, Wendisch VF, Wiegräbe I, Tauch A. 2003. The complete *Corynebacterium glutamicum* ATCC 13032 genome sequence and its impact on the production of L-aspartate-derived amino acids and vitamins. J Biotechnol 104:5-25.

19. Studier FW, Moffatt BA. 1986. Use of bacteriophage T7 RNA polymerase to direct selective high-level expression of cloned genes. J Mol Biol 189:113-130.
