# Supplementary material for: Deciphering the Rules Underlying Xenogeneic Silencing and Counter-Silencing of Lsr2-like Proteins Using CgpS of Corynebacterium glutamicum as a Model
Source: mBio. 2020 Feb 4;11(1):e02273-19. doi: 10.1128/mBio.02273-19 (PMC7002338; doi:10.1128/mBio.02273-19)
Supplement: TABLE S2 [file mBio.02273-19-st002.docx]

**SUPPLEMENTAL MATERIAL**

**SUPPLEMENTARY INFORMATION: TABLE S2**

**TABLE S2: Strains, plasmids, oligonucleotides and DNA sequences**

**Table S2A: Strains used in this study.**

| Strain | Relevant characteristics | Reference |
| --- | --- | --- |
| *E. coli* |  |  |
| DH5α | F^-^ Φ80*lacZ*ΔM15 Δ(*lacZYA*-*argF*) U169 *recA1* *endA1* *hsdR17*(r_k_^-^, m_k_^+^) *phoA* *supE44* *thi-1* *gyrA96 relA1* λ^-^, strain used for cloning procedures | Invitrogen |
| BL21(DE3) | \| F^−^ *ompT hsdS*B(rB− mB−) *gal dcm* BL21(DE3), \| \| --- \|   strain used for protein production | (1) |
| *Vibrio natrigens* |  |  |
| *Vibrio natriegens* Vmax^TM^ |  | Synthetic Genomics, San Diego, CA, USA |
| *C. glutamicum* |  |  |
| ATCC 13032 | Biotin-auxotrophic wild type | (2) |
| Δ*gntR1/2* | Derivate of ATCC 13032 with in-frame deletions of genes *gntR1* (cg2783) and *gntR2* (cg1935) | (3) |
| Δphage (MB001) | Derivate of ATCC 13032 with deletion of prophages CGP1 (cg1507-cg1524), CGP2 (cg1746-cg1752), and CGP3 (cg1890-cg2071) | (4) |
| Δphage::P*_cgpS_*-*cgpS* | Derivate of Δphage (MB001) with deletion of prophages CGP1 (cg1507-cg1524), CGP2 (cg1746-cg1752), and CGP3 (cg1890-cg2071) and re-integrated cgpS together with its native promoter in the intergenic region of cg1199-cg1201 | This work |

**Table S2B: Plasmids from other studies used in this study.**

| Plasmid | Relevant characteristics | Source or reference |
| --- | --- | --- |
| pJC1 | *Kan^R^*, *Amp^R^*; *oriV_C.g_*_._ , *oriV_E.c._* (*C. glutamicum*/*E. coli* shuttle vector) | (5) |
| pJC1-*venus*-term | *Kan^R^*, pJC1 derivative carrying the *venus* coding sequence followed by a terminator sequence of *Bacillus subtilis* | (4) |
| pJC1-P*_lysin_*-*e2-crimson* | *Kan^R^*; pJC1 derivative containing a 250 bp promoter region of the *lys* gene (cg1974) and the first 30 bp of the coding sequence fused to *e2-crimson* gene via a linker containing a stop codon and a RBS | (6) |
| pK19-*mobsacB* | *Kan^R^*; plasmid for allelic exchange in *C. glutamicum*; (*oriT*, *oriVE.c*_._, *sacB*, *lacZα*) | (7) |
| pAN6 | *Kan^R^*; *C. glutamicum*/*E. coli* shuttle vector for gene expression under control of the P*_tac_* promoter; (P*_tac_*, *lacIq*, pBL1 *oriVC.g.*, pUC18 *oriVE.c.*) | (3) |
| pAN6-*cgpS*-Strep | *Kan^R^*; pAN6 derivative containing the *cgpS* gene fused C-terminal without stop codon to a sequence encoding a Strep-tag | (8) |
| pET16b | *Amp^R^*; vector for overexpression of genes in *E. coli*, adding a C-terminal hexahistidine affinity tag to the synthesized protein (pBR322 *oriVE.c.*, P*_T7_*, *lacI* ) | Novagen |
| pET16b-*gntR1* | *Amp^R^*; pET16b derivative for overproduction of GntR1 with an N-terminal decahistidine tag. | (3) |

**Table S2C: Plasmids constructed in this work.** Oligonucleotide pairs, which were used for PCR, are given as numbers (Table S2D). DNA templates are indicated in brackets behind the oligonucleotides. The used backbones including the restriction enzymes used for linearization are listed behind (*). Information about region of CgpS bound area and positions of maximal CgpS binding coverage were based on previous studies from Pfeifer and colleagues (8).

| Plasmid | Construction | Relevant characteristics |
| --- | --- | --- |
| Phage promoter-based constructs | | |
| pJC1-P_cg1897_-*venus* | Gibson assembly: 459/460 (*C. glutamicum* genome) and 115/116 (pJC1-*venus*-term) into pJC1-*venus*-term *BamHI *BcuI | *Kan^R^*; pJC1-*venus*-term derivative carrying the CgpS bound area of the promoter of cg1897 (468 bp) and the first 30 bp of the coding sequence fused to the reporter gene *venus* via a linker containing a stop codon and an artificial RBS. |
| pJC1-P_cg1897_::GntR_BS_pos0-*venus* | Gibson assembly: 459/394 (pJC1-P_cg1897_-*venus*) and 393/116 (pJC1-P_cg1897_-*venus*) into pJC1-*venus*-term *BamHI *BcuI | *Kan^R^*; pJC1-P_cg1897_-*venus* derivative with an inserted GntR BS directly upstream of the position of maximal CgpS binding. |
| pJC1-P_cg1936_-*venus* | Gibson assembly: 449/450 (*C. glutamicum* genome) and 115/116 (pJC1-*venus*-term) into pJC1-*venus*-term *BamHI *BcuI | *Kan^R^*; pJC1-*venus*-term derivative carrying the CgpS bound area of the promoter of cg1936 (676 bp) and the first 30 bp of the coding sequence fused to the reporter gene *venus* via a linker containing a stop codon and an artificial RBS. |
| pJC1-P_cg1936_::GntR_BS_pos0-*venus* | Gibson assembly: 449/452 (pJC1-P_cg1936_-*venus*) and 451/116 (pJC1-P_cg1936_-*venus*) into pJC1-*venus*-term *BamHI *BcuI | *Kan^R^*; pJC1-P_cg1936_-*venus* derivative with an inserted GntR BS directly upstream of the position of maximal CgpS binding. |
| pJC1-P_cg1940_-*venus* | Gibson assembly: 444/445 (*C. glutamicum* genome) and 115/116 (pJC1-*venus*-term) into pJC1-*venus*-term *BamHI *BcuI | *Kan^R^*; pJC1-*venus*-term derivative carrying the CgpS bound area of the promoter of cg1940 (563 bp) and the first 30 bp of the coding sequence fused to the reporter gene *venus* via a linker containing a stop codon and an artificial RBS. |
| pJC1-P_cg1940_::GntR_BS_pos0-*venus* | Gibson assembly: 444/447 (pJC1-P_cg1940_-*venus*) and 446/116 (pJC1-P_cg1940_-*venus*) into pJC1-*venus*-term *BamHI *BcuI | *Kan^R^*; pJC1-P_cg1940_-*venus* derivative with an inserted GntR BS directly upstream of the position of maximal CgpS binding. |
| pJC1-P_cg1955_-*venus* | Gibson assembly: 387/388 (*C. glutamicum* genome) and 115/116 (pJC1-*venus*-term) into pJC1-*venus*-term *BamHI *BcuI | *Kan^R^*; pJC1-*venus*-term derivative carrying the CgpS bound area of the promoter of cg1955 (516 bp) and the first 30 bp of the coding sequence fused to the reporter gene *venus* via a linker containing a stop codon and an artificial RBS. |
| pJC1-P_cg1955_::GntR_BS_pos0-*venus* | Gibson assembly: 387/390 (pJC1-P_cg1955_-*venus*) and 389/116 (pJC1-P_cg1955_-*venus*) into pJC1-*venus*-term *BamHI *BcuI | *Kan^R^*; pJC1-P_cg1955_-*venus* derivative with an inserted GntR BS directly upstream of the position of maximal CgpS binding. |
| pJC1-P*_priP_*-*venus* | Gibson assembly: 200/201 (*C. glutamicum* genome) and 115/116 (pJC1-*venus*-term) into pJC1-*venus*-term *BamHI *BcuI | *Kan^R^*; pJC1-*venus*-term derivative carrying the CgpS bound area of the promoter of P*_priP_* (611 bp) and the first 30 bp of the coding sequence fused to the reporter gene *venus* via a linker containing a stop codon and an artificial RBS. |
| pJC1-P*_priP_*::GntR_BS_pos0-*venus* | Gibson assembly: 200/191 (pJC1-P*_priP_*-*venus*) and 190/116 (pJC1-P*_priP_*-*venus*) into pJC1-*venus*-term *BamHI *BcuI | *Kan^R^*; pJC1-P*_priP_*-*venus* derivative with an inserted GntR BS directly upstream of the position of maximal CgpS binding. |
| pJC1-P*_lys_*-*venus* | Gibson assembly: 117/114 (*C. glutamicum* genome) and 115/116 (pJC1-*venus*-term) into pJC1-*venus*-term *BamHI *BcuI | *Kan^R^*; pJC1-*venus*-term derivative carrying the CgpS bound area of the promoter of the *lys* gene (cg1974) (444 bp) and the first 30 bp of the coding sequence fused to the reporter gene *venus* via a linker containing a stop codon and an artificial RBS. |
| pJC1-P*_lys_*::GntR_BS_pos-100-*venus* | Gibson assembly: 117/119 (pJC1-P*_lys_*-*venus*) and 118/116 (pJC1-P*_lys_*-*venus*) into pJC1-*venus*-term *BamHI *BcuI | *Kan^R^*; pJC1-P*_lys_*-*venus* derivative with an inserted GntR BS 100 bp upstream of the position of maximal CgpS binding. |
| pJC1-P*_lys_*::GntR_BS_pos-50-*venus* | Gibson assembly: 117/114 (pEK-A2-P*_lys_*::GntR_BS-50 bp upstream) and 115/116 (pJC1-*venus*-term) into pJC1-*venus*-term *BamHI *BcuI | *Kan^R^*; pJC1-P*_lys_*-*venus* derivative with an inserted GntR BS 50 bp upstream of the position of maximal CgpS binding. |
| pJC1-P*_lys_*::GntR_BS_pos-25-*venus* | Gibson assembly: 117/178 (pJC1-P*_lys_*-*venus*) and 177/116 (pJC1-P*_lys_*-*venus*) into pJC1-*venus*-term *BamHI *BcuI | *Kan^R^*; pJC1-P*_lys_*-*venus* derivative with an inserted GntR BS 25 bp upstream of the position of maximal CgpS binding. |
| pJC1-P*_lys_*::GntR_BS_pos-20-*venus* | Gibson assembly: 117/168 (pJC1-P*_lys_*-*venus*) and 167/116 (pJC1-P*_lys_*-*venus*) into pJC1-*venus*-term *BamHI *BcuI | *Kan^R^*; pJC1-P*_lys_*-*venus* derivative with an inserted GntR BS 20 bp upstream of the position of maximal CgpS binding. |
| pJC1-P*_lys_*::GntR_BS_pos-15-*venus* | Gibson assembly: 117/176 (pJC1-P*_lys_*-*venus*) and 175/116 (pJC1-P*_lys_*-*venus*) into pJC1-*venus*-term *BamHI *BcuI | *Kan^R^*; pJC1-P*_lys_*-*venus* derivative with an inserted GntR BS 15 bp upstream of the position of maximal CgpS binding. |
| pJC1-P*_lys_*::GntR_BS_pos-10-*venus* | Gibson assembly: 117/174 (pJC1-P*_lys_*-*venus*) and 173/116 (pJC1-P*_lys_*-*venus*) into pJC1-*venus*-term *BamHI *BcuI | *Kan^R^*; pJC1-P*_lys_*-*venus* derivative with an inserted GntR BS 10 bp upstream of the position of maximal CgpS binding. |
| pJC1-P*_lys_*::GntR_BS_pos-5-*venus* | Gibson assembly: 117/172 (pJC1-P*_lys_*-*venus*) and 171/116 (pJC1-P*_lys_*-*venus*) into pJC1-*venus*-term *BamHI *BcuI | *Kan^R^*; pJC1-P*_lys_*-*venus* derivative with an inserted GntR BS 5 bp upstream of the position of maximal CgpS binding. |
| pJC1-P*_lys_*::GntR_BS_pos-4-*venus* | Gibson assembly: 117/244 (pJC1-P*_lys_*-*venus*) and 243/116 (pJC1-P*_lys_*-*venus*) into pJC1-*venus*-term *BamHI *BcuI | *Kan^R^*; pJC1-P*_lys_*-*venus* derivative with an inserted GntR BS 4 bp upstream of the position of maximal CgpS binding. |
| pJC1-P*_lys_*::GntR_BS_pos-3-*venus* | Gibson assembly: 117/246 (pJC1-P*_lys_*-*venus*) and 245/116 (pJC1-P*_lys_*-*venus*) into pJC1-*venus*-term *BamHI *BcuI | *Kan^R^*; pJC1-P*_lys_*-*venus* derivative with an inserted GntR BS 3 bp upstream of the position of maximal CgpS binding. |
| pJC1-P*_lys_*::GntR_BS_pos-2-*venus* | Gibson assembly: 117/248 (pJC1-P*_lys_*-*venus*) and 247/116 (pJC1-P*_lys_*-*venus*) into pJC1-*venus*-term *BamHI *BcuI | *Kan^R^*; pJC1-P*_lys_*-*venus* derivative with an inserted GntR BS 2 bp upstream of the position of maximal CgpS binding. |
| pJC1-P*_lys_*::GntR_BS_pos-1-*venus* | Gibson assembly: 117/250 (pJC1-P*_lys_*-*venus*) and 249/116 (pJC1-P*_lys_*-*venus*) into pJC1-*venus*-term *BamHI *BcuI | *Kan^R^*; pJC1-P*_lys_*-*venus* derivative with an inserted GntR BS 1 bp upstream of the position of maximal CgpS binding. |
| pJC1-P*_lys_*_CS_0-*venus* | Gibson assembly: 117/125 (pJC1-P*_lys_*-*venus*) and 124/116 (pJC1-P*_lys_*-*venus*) into pJC1-*venus*-term *BamHI *BcuI | *Kan^R^*; pJC1-P*_lys_*-*venus* derivative with an inserted GntR BS directly upstream of the position of maximal CgpS binding. |
| pJC1-P*_lys_*::GntR_BS_pos+1-*venus* | Gibson assembly: 117/212 (pJC1-P*_lys_*-*venus*) and 211/116 (pJC1-P*_lys_*-*venus*) into pJC1-*venus*-term *BamHI *BcuI | *Kan^R^*; pJC1-P*_lys_*-*venus* derivative with an inserted GntR BS 1 bp downstream of the position of maximal CgpS binding. |
| pJC1-P*_lys_*::GntR_BS_pos+2-*venus* | Gibson assembly: 117/252 (pJC1-P*_lys_*-*venus*) and 251/116 (pJC1-P*_lys_*-*venus*) into pJC1-*venus*-term *BamHI *BcuI | *Kan^R^*; pJC1-P*_lys_*-*venus* derivative with an inserted GntR BS 2 bp downstream of the position of maximal CgpS binding. |
| pJC1-P*_lys_*::GntR_BS_pos+3-*venus* | Gibson assembly: 117/254 (pJC1-P*_lys_*-*venus*) and 253/116 (pJC1-P*_lys_*-*venus*) into pJC1-*venus*-term *BamHI *BcuI | *Kan^R^*; pJC1-P*_lys_*-*venus* derivative with an inserted GntR BS 3 bp downstream of the position of maximal CgpS binding. |
| pJC1-P*_lys_*::GntR_BS_pos+4-*venus* | Gibson assembly: 117/256 (pJC1-P*_lys_*-*venus*) and 255/116 (pJC1-P*_lys_*-*venus*) into pJC1-*venus*-term *BamHI *BcuI | *Kan^R^*; pJC1-P*_lys_*-*venus* derivative with an inserted GntR BS 4 bp downstream of the position of maximal CgpS binding. |
| pJC1-P*_lys_*::GntR_BS_pos+5-*venus* | Gibson assembly: 117/180 (pJC1-P*_lys_*-*venus*) and 179/116 (pJC1-P*_lys_*-*venus*) into pJC1-*venus*-term *BamHI *BcuI | *Kan^R^*; pJC1-P*_lys_*-*venus* derivative with an inserted GntR BS 5 bp downstream of the position of maximal CgpS binding. |
| pJC1-P*_lys_*::GntR_BS_pos+10-*venus* | Gibson assembly: 117/182 (pJC1-P*_lys_*-*venus*) and 181/116 (pJC1-P*_lys_*-*venus*) into pJC1-*venus*-term *BamHI *BcuI | *Kan^R^*; pJC1-P*_lys_*-*venus* derivative with an inserted GntR BS 10 bp downstream of the position of maximal CgpS binding. |
| pJC1-P*_lys_*::GntR_BS_pos+15-*venus* | Gibson assembly: 117/184 (pJC1-P*_lys_*-*venus*) and 183/116 (pJC1-P*_lys_*-*venus*) into pJC1-*venus*-term *BamHI *BcuI | *Kan^R^*; pJC1-P*_lys_*-*venus* derivative with an inserted GntR BS 15 bp downstream of the position of maximal CgpS binding. |
| pJC1-P*_lys_*::GntR_BS_pos+20-*venus* | Gibson assembly: 117/186 (pJC1-P*_lys_*-*venus*) and 185/116 (pJC1-P*_lys_*-*venus*) into pJC1-*venus*-term *BamHI *BcuI | *Kan^R^*; pJC1-P*_lys_*-*venus* derivative with an inserted GntR BS 20 bp downstream of the position of maximal CgpS binding. |
| pJC1-P*_lys_*::GntR_BS_pos+25-*venus* | Gibson assembly: 117/121 (pJC1-P*_lys_*-*venus*) and 120/116 (pJC1-P*_lys_*-*venus*) into pJC1-*venus*-term *BamHI *BcuI | *Kan^R^*; pJC1-P*_lys_*-*venus* derivative with an inserted GntR BS 25 bp downstream of the position of maximal CgpS binding. |
| pJC1-P*_lys_*::GntR_BS_pos+50-*venus* | Gibson assembly: 117/188 (pJC1-P*_lys_*-*venus*) and 187/116 (pJC1-P*_lys_*-*venus*) into pJC1-*venus*-term *BamHI *BcuI | *Kan^R^*; pJC1-P*_lys_*-*venus* derivative with an inserted GntR BS 50 bp downstream of the position of maximal CgpS binding. |
| pJC1-P*_lys_*::control_sequence_1_pos0-*venus* | Gibson assembly: 117/380 (pJC1-P*_lys_*-*venus*) and 379/116 (pJC1-P*_lys_*-*venus*) into pJC1-*venus*-term *BamHI *BcuI | *Kan^R^*; pJC1-P*lys*-*venus* derivative with an inserted control sequence 1 (CATTAATGATAATGC) directly upstream of the position of maximal CgpS binding. |
| pJC1-P*_lys_*::control_sequence_2_pos0-*venus* | Gibson assembly: 117/382 (pJC1-P*_lys_*-*venus*) and 381/116 (pJC1-P*_lys_*-*venus*) into pJC1-*venus*-term *BamHI *BcuI | *Kan^R^*; pJC1-P*_lys_*-*venus* derivative with an inserted control sequence 2 (TATGATAGTAGCAAT) directly upstream of the position of maximal CgpS binding. |
| pJC1-P*_lys_*(5´∆300bp)-*venus* | Gibson assembly: 385/116 (pJC1-P*_lys_*-*venus*) into pJC1-*venus*-term *BamHI *BcuI | *Kan^R^*; pJC1-P*_lys_*-*venus* derivative with a 300 bp truncation of the 5´-promoter region. |
| pJC1-P*_lys_*(5´∆350bp)-*venus* | Gibson assembly: 386/116 (pJC1-P*_lys_*-*venus*) into pJC1-*venus*-term *BamHI *BcuI | *Kan^R^*; pJC1-P*lys*-*venus* derivative with a 350 bp truncation of the 5´-promoter region. |
| pJC1-P*_lys_*(5´∆300bp)_CS_0-*venus* | Gibson assembly: 385/116 (pJC1-P*_lys_*_CS_0-*venus*) into pJC1-*venus*-term *BamHI *BcuI | *Kan^R^*; pJC1-P*_lys_*_CS_0-*venus* derivative with a 300 bp truncation of the 5´-promoter region. |
| pJC1-P*_lys_*(5´∆350bp)_CS_0-*venus* | Gibson assembly: 386/116 (pJC1-P*_lys_*_CS_0-*venus*) into pJC1-*venus*-term *BamHI *BcuI | *Kan^R^*; pJC1-P*_lys_*_CS_0-*venus* derivative with a 350 bp truncation of the 5´-promoter region. |
| pJC1-P_cg1977_-*venus* | Gibson assembly: 435/436 (*C. glutamicum* genome) and 115/116 (pJC1-*venus*-term) into pJC1-*venus*-term *BamHI *BcuI | *Kan^R^*; pJC1-*venus*-term derivative carrying the CgpS bound area of the promoter of cg1977 (653 bp) and the first 30 bp of the coding sequence fused to the reporter gene *venus* via a linker containing a stop codon and an artificial RBS. |
| pJC1-P_cg1977_::GntR_BS_pos0-*venus* | Gibson assembly: 435/438 (pJC1-P_cg1977_-*venus*) and 437/116 (pJC1-P_cg1977_-*venus*) into pJC1-*venus*-term *BamHI *BcuI | *Kan^R^*; pJC1-P_cg1977_-*venus* derivative with an inserted GntR BS directly upstream of the position of maximal CgpS binding. |
| pJC1-P_cg1999_-*venus* | Gibson assembly: 395/396 (*C. glutamicum* genome) and 115/116 (pJC1-*venus*-term) into pJC1-*venus*-term *BamHI *BcuI | *Kan^R^*; pJC1-*venus*-term derivative carrying the CgpS bound area of the promoter of cg1999 (448 bp) and the first 30 bp of the coding sequence fused to the reporter gene *venus* via a linker containing a stop codon and an artificial RBS. |
| pJC1-P_cg1999_::GntR_BS_pos-30-*venus* | Gibson assembly: 395/575 (pJC1-P_cg1999_-*venus*) and 574/116 (pJC1-P_cg1999_-*venus*) into pJC1-*venus*-term *BamHI *BcuI | *Kan^R^*; pJC1-P_cg1999_-*venus* derivative with an inserted GntR BS 30 bp upstream of the position of maximal CgpS binding. |
| pJC1-P_cg1999_::GntR_BS_pos-20-*venus* | Gibson assembly: 395/577 (pJC1-P_cg1999_-*venus*) and 576/116 (pJC1-P_cg1999_-*venus*) into pJC1-*venus*-term *BamHI *BcuI | *Kan^R^*; pJC1-P_cg1999_-*venus* derivative with an inserted GntR BS 20 bp upstream of the position of maximal CgpS binding. |
| pJC1-P_cg1999_::GntR_BS_pos-10-*venus* | Gibson assembly: 395/579 (pJC1-P_cg1999_-*venus*) and 578/116 (pJC1-P_cg1999_-*venus*) into pJC1-*venus*-term *BamHI *BcuI | *Kan^R^*; pJC1-P_cg1999_-*venus* derivative with an inserted GntR BS 10 bp upstream of the position of maximal CgpS binding. |
| pJC1-P_cg1999_::GntR_BS_pos-5-*venus* | Gibson assembly: 395/573 (pJC1-P_cg1999_-*venus*) and 572/116 (pJC1-P_cg1999_-*venus*) into pJC1-*venus*-term *BamHI *BcuI | *Kan^R^*; pJC1-P_cg1999_-*venus* derivative with an inserted GntR BS 5 bp upstream of the position of maximal CgpS binding. |
| pJC1-P_cg1999_::GntR_BS_pos0-*venus* | Gibson assembly: 395/398 (pJC1-P_cg1999_-*venus*) and 397/116 (pJC1-P_cg1999_-*venus*) into pJC1-*venus*-term *BamHI *BcuI | *Kan^R^*; pJC1-P_cg1999_-*venus* derivative with an inserted GntR BS directly upstream of the position of maximal CgpS binding. |
| pJC1-P_cg1999_::GntR_BS_pos+5-*venus* | Gibson assembly: 395/571 (pJC1-P_cg1999_-*venus*) and 570/116 (pJC1-P_cg1999_-*venus*) into pJC1-*venus*-term *BamHI *BcuI | *Kan^R^*; pJC1-P_cg1999_-*venus* derivative with an inserted GntR BS 5 bp downstream of the position of maximal CgpS binding. |
| pJC1-P_cg1999_::GntR_BS_pos+10-*venus* | Gibson assembly: 395/581 (pJC1-P_cg1999_-*venus*) and 580/116 (pJC1-P_cg1999_-*venus*) into pJC1-*venus*-term *BamHI *BcuI | *Kan^R^*; pJC1-P_cg1999_-*venus* derivative with an inserted GntR BS 10 bp downstream of the position of maximal CgpS binding. |
| pJC1-P_cg2014_-*venus* | Gibson assembly: 439/440 (*C. glutamicum* genome) and 115/116 (pJC1-*venus*-term) into pJC1-*venus*-term *BamHI *BcuI | *Kan^R^*; pJC1-*venus*-term derivative carrying the CgpS bound area of the promoter of cg2014 (545 bp) and the first 30 bp of the coding sequence fused to the reporter gene *venus* via a linker containing a stop codon and an artificial RBS. |
| pJC1-P_cg2014_::GntR_BS_pos0-*venus* | Gibson assembly: 439/442 (pJC1-P_cg2014_-*venus*) and 441/116 (pJC1-P_cg2014_-*venus*) into pJC1-*venus*-term *BamHI *BcuI | *Kan^R^*; pJC1-P_cg2014_-*venus* derivative with an inserted GntR BS directly upstream of the position of maximal CgpS binding. |
| pJC1-P_cg2020_-*venus* | Gibson assembly: 408/409 (*C. glutamicum* genome) and 115/116 (pJC1-*venus*-term) into pJC1-*venus*-term *BamHI *BcuI | *Kan^R^*; pJC1-*venus*-term derivative carrying the CgpS bound area of the promoter of cg2020 (390 bp) and the first 30 bp of the coding sequence fused to the reporter gene *venus* via a linker containing a stop codon and an artificial RBS. |
| pJC1-P_cg2020_::GntR_BS_pos0-*venus* | Gibson assembly: 408/411 (pJC1-P_cg2020_-*venus*) and 410/116 (pJC1-P_cg2020_-*venus*) into pJC1-*venus*-term *BamHI *BcuI | *Kan^R^*; pJC1-P_cg2020_-*venus* derivative with an inserted GntR BS directly upstream of the position of maximal CgpS binding. |
| pJC1-P_cg2022_-*venus* | Gibson assembly: 416/417 (*C. glutamicum* genome) and 115/116 (pJC1-*venus*-term) into pJC1-*venus*-term *BamHI *BcuI | *Kan^R^*; pJC1-*venus*-term derivative carrying the CgpS bound area of the promoter of cg2022 (309 bp) and the first 30 bp of the coding sequence fused to the reporter gene *venus* via a linker containing a stop codon and an artificial RBS. |
| pJC1-P_cg2022_::GntR_BS_pos0-*venus* | Gibson assembly: 416/419 (pJC1-P_cg2022_-*venus*) and 418/116 (pJC1-P_cg2022_-*venus*) into pJC1-*venus*-term *BamHI *BcuI | *Kan^R^*; pJC1-P_cg2022_-*venus* derivative with an inserted GntR BS directly upstream of the position of maximal CgpS binding. |
| pJC1-P_cg2032_-*venus* | Gibson assembly: 412/413 (*C. glutamicum* genome) and 115/116 (pJC1-*venus*-term) into pJC1-*venus*-term *BamHI *BcuI | *Kan^R^*; pJC1-*venus*-term derivative carrying the CgpS bound area of the promoter of cg2032 (490 bp) and the first 30 bp of the coding sequence fused to the reporter gene *venus* via a linker containing a stop codon and an artificial RBS. |
| pJC1-P_cg2032_::GntR_BS_pos0-*venus* | Gibson assembly: 412/415 (pJC1-P_cg2032_-*venus*) and 414/116 (pJC1-P_cg2032_-*venus*) into pJC1-*venus*-term *BamHI *BcuI | *Kan^R^*; pJC1-P_cg2032_-*venus* derivative with an inserted GntR BS directly upstream of the position of maximal CgpS binding. |
| Synthetic P_cg1999_ promoter variants | | |
| pJC1-P_cg1999__A-T/G-C-*venus* | Gibson assembly: 501/505 (pJC1-P_cg1999__A-T/G-C) and 115/116 (pJC1-*venus*-term) into pJC1-*venus*-term *BamHI *BcuI | *Kan^R^*; pJC1-*venus*-term derivative carrying a synthetic promoter based on a 50 bp region of P_cg1999_ containing core promoter elements (TSS, -10 and -35 box), the identified binding motif and the nucleotide associated with maximal CgpS coverage. GC-profile of P_cg1999_ was maintain in the adjacent flanks but A/T and G/C were swapped. The promoter was fused to the reporter gene *venus* via a linker containing a stop codon and an artificial RBS. The distance between TSS, start of coding region and artificial 30 bp coding region as well as the linker containing the RBS were consistent with the template sequence of P_cg1999_. |
| pJC1-P_cg1999__rand-*venus* | Gibson assembly: 497/498 (pEX-K168-Synthetic-sequence1) and 115/116 (pJC1-*venus*-term) into pJC1-*venus*-term *BamHI *BcuI | *Kan^R^*; pJC1-*venus*-term derivative carrying a synthetic promoter based on a 50 bp region of P_cg1999_ containing core promoter elements (TSS, -10 and -35 box), the identified binding motif and the nucleotide associated with maximal CgpS coverage. The adjacent sequences were randomized and fused to the reporter gene *venus* via a linker containing a stop codon and an artificial RBS. The distance between TSS, start of coding region and artificial 30 bp coding region as well as the linker containing the RBS were consistent with the template sequence of P_cg1999_. |
| Synthetic P*_lys_* promoter variants | | |
| pJC1-P*_lys_*_A-T/G-C_70bp-*venus* | Gibson assembly: 560/601 (gene strand P*_lys_*_A-T/G-C) and 115/116 (pJC1-*venus*-term) into pJC1-*venus*-term *BamHI *BcuI | *Kan^R^*; pJC1-*venus*-term derivative carrying a synthetic promoter based on a 70 bp region of P*_lys_* containing core promoter elements (TSS, -10 and -35 box). GC-profile of P*_lys_* was maintain in the adjacent flanks but A/T and G/C were swapped. The promoter was fused to the reporter gene *venus* via a linker containing a stop codon and an artificial RBS. The distance between TSS, start of coding region and artificial 30 bp coding region as well as the linker containing the RBS were consistent with the template sequence of P*_lys_*. |
| pJC1-P*_lys_*_A-T/G-C_80bp-*venus* | Gibson assembly: 560/618 (pJC1-P*_lys_*_A-T/G-C_70bp-*venus*) and 617/116 (pJC1-P*_lys_*_A-T/G-C_70bp-*venus*) into pJC1-*venus*-term *BamHI *BcuI | *Kan^R^*; pJC1-*venus*-term derivative carrying a synthetic promoter based on a 80 bp region of P*_lys_* containing core promoter elements (TSS, -10 and -35 box) and the identified binding motif. GC-profile of P*_lys_* was maintain in the adjacent flanks but A/T and G/C were swapped. The promoter was fused to the reporter gene *venus* via a linker containing a stop codon and an artificial RBS. The distance between TSS, start of coding region and artificial 30 bp coding region as well as the linker containing the RBS were consistent with the template sequence of P*_lys_*. |
| pJC1-P*_lys_*_A-T/G-C_70bp-*venus* | Gibson assembly: 560/601 (gene strand P*_lys_*_A-T/G-C) and 115/116 (pJC1-*venus*-term) into pJC1-*venus*-term *BamHI *BcuI | *Kan^R^*; pJC1-*venus*-term derivative carrying a synthetic promoter based on a 100 bp region of P*_lys_* containing core promoter elements (TSS, -10 and -35 box), the identified binding motif and the nucleotide associated with maximal CgpS coverage (8). GC-profile of P*_lys_* was maintain in the adjacent flanks but A/T and G/C were swapped. The promoter was fused to the reporter gene *venus* via a linker containing a stop codon and an artificial RBS. The distance between TSS, start of coding region and artificial 30 bp coding region as well as the linker containing the RBS were consistent with the template sequence of P*_lys_*. |
| Further reporter constructs | | |
| pJC1-P*_gntK_*-*venus* | Gibson assembly: 203/204 (*C. glutamicum* genome) and 115/116 (pJC1-*venus*-term) into pJC1-*venus*-term *BamHI *BcuI | *Kan^R^*; pJC1-*venus*-term derivative carrying the P*_gntK_* promoter (307 bp) (P_cg2732_) and the first 30 bp of the coding sequence fused to the reporter gene *venus* via a linker containing a stop codon and an artificial RBS. |
| pJC1-P*_gntK_*-*e2-crimson* | Gibson assembly: 203/204 (*C. glutamicum* genome) and 259/260 (pJC1-P*_lysin_*-*e2-crimson*) into pJC1-*venus*-term *BamHI *BcuI | *Kan^R^*; pJC1-*venus*-term derivative carrying the P*_gntK_* promoter (307 bp) (P_cg2732_) and the first 30 bp of the coding sequence fused to the reporter gene *e2-crimson* via a linker containing a stop codon and an artificial RBS. |
| GntR-dependent toggle | | |
| pJC1-P*_lys_*_CS_0-*venus*-T-P*_gntK_*-*e2-crimson* | Gibson assembly: 117/263 (pJC1-P*_lys_*_CS_0-*venus*) and 261/262 (pJC1-P*_gntK_*-*e2-crimson*) into pJC1-*venus*-term *BamHI *BcuI | *Kan^R^*; pJC1-*venus*-term derivative carrying the construct P*_lys_*_CS_0-*venus* and the oppositely oriented P*_gntK_*-*e2-crimson* construct. |
| Templates used for preparation of DNA fragments for surface plasmon resonance analysis | | |
| pJC1-P_cg3336_ | Gibson assembly: 516/517 (*C. glutamicum* genome) into pJC1-*venus*-term *BamHI *BcuI | *Kan^R^*; pJC1-*venus*-term derivative carrying a 1238 bp region covering the promoter region of cg3336 |
| pJC1-P_cg1999_ | Gibson assembly: 520/521 (*C. glutamicum* genome) into pJC1-*venus*-term *BamHI *BcuI | *Kan^R^*; pJC1-*venus*-term derivative carrying a 1363 bp region covering the promoter region of c1999 |
| pJC1-P_cg1999__A-T/G-C | Gibson assembly: 501/503 (pEX-K168-Cg1999-AT-GCSwitch) and 502/504 (pEX-K168-Cg1999-AT-GCSwitch) into pJC1-*venus*-term *BamHI *BcuI | *Kan^R^*; pJC1-*venus*-term derivative carrying the sequence P_cg1999__A-T/G-C encoded in pEX-K168-Cg1999-AT-GCSwitch (Table S2H) with nucleotide exchanges to ATG at positions 331-333 |
| pJC1-P*_lys_*_CS_0 | Gibson assembly: 117/125 (*C. glutamicum* genome) and 124/404 (*C. glutamicum* genome) into pJC1-*venus*-term *BamHI *BcuI | *Kan^R^*; pJC1-*venus*-term derivative carrying the CgpS bound area of the promoter of *lys* (cg1974) (444 bp) with an inserted GntR BS directly upstream of the position of maximal CgpS binding and the first 179 bp of the *lys* gene. |
| *cgpS* integration plasmid | | |
| pK19*mobsacB*-1199_1201-P*_cgpS_*-*cgpS* | Gibson assembly: E227/E228 (*C. glutamicum* genome), E245/E115 (*C. glutamicum* genome) and E229/E230 (*C. glutamicum* genome) into pK19*mobsacB* *HindIII *EcoRI | *Kan^R^*; pK19-*mobsacB* derivative for the re-integration of *cgpS* and its native promoter (500+351bp) into the intergenic region of cg1199-cg1201 (500 bp upstream and downstream flanking region). |

**Table S2D: Oligonucleotides used in this study for plasmid constructions.**

| Oligonucleotide number | Sequence (5´🡪3´) |
| --- | --- |
| 114 | TGATATCTCCTTCTTAAAGTTCAATTTTTCGGCATTGCGCCTTTAATCGC |
| 115 | TGAACTTTAAGAAGGAGATATCATATGGTGAGCAAGGGCGAGGAG |
| 116 | AAAACGACGGCCAGTACTAGTTACTTGTACAGCTCGTCCATGCC |
| 117 | AGCGACGCCGCAGGGGGATCCGCTCAAGGAAGAGTTCTTCATTGGTC |
| 118 | GCCTTTATGATAGTACCAATTCGAGAACTGGGTGTAGTGATTTCTG |
| 119 | GTTCTCGAATTGGTACTATCATAAAGGCTTTTTCTCTCATGACCTACCC |
| 120 | GGGAACATTATGATAGTACCAATTAAACGGGTAAAGGTAAAGGACAAACG |
| 121 | CCCGTTTAATTGGTACTATCATAATGTTCCCTGGATACTACAAATTAAAC |
| 124 | GATACTAGAGTTATGATAGTACCAATTTAATTTGTAGTATCCAGGGAAC |
| 125 | AAATTAAATTGGTACTATCATAACTCTAGTATCTTATTAATTTCTGTTAC |
| 167 | CAGATATGATAGTACCAATAATTAATAAGATACTAGAGTTTAATTTGTAG |
| 168 | CTTATTAATTATTGGTACTATCATATCTGTTACTATTAACATATAGGTTT |
| 171 | GATACTTATGATAGTACCAATAGAGTTTAATTTGTAGTATCCAGGGAAC |
| 172 | CTCTATTGGTACTATCATAAGTATCTTATTAATTTCTGTTACTATTAAC |
| 173 | GTATGATAGTACCAATATACTAGAGTTTAATTTGTAGTATCCAGGG |
| 174 | GTATATTGGTACTATCATACTTATTAATTTCTGTTACTATTAACATATAG |
| 175 | ATTATATGATAGTACCAATATAAGATACTAGAGTTTAATTTGTAGTATCC |
| 176 | CTTATATTGGTACTATCATATAATTTCTGTTACTATTAACATATAGG |
| 177 | TATATGATAGTACCAATACAGAAATTAATAAGATACTAGAGTTTAATTTG |
| 178 | ATTTCTGTATTGGTACTATCATATACTATTAACATATAGGTTTTTTAAAG |
| 179 | GTTTAATTATGATAGTACCAATTTGTAGTATCCAGGGAACATTAAACGG |
| 180 | AAATTGGTACTATCATAATTAAACTCTAGTATCTTATTAATTTCTGTTAC |
| 181 | GTATATGATAGTACCAATGTATCCAGGGAACATTAAACGGGTAAAG |
| 182 | TACATTGGTACTATCATATACAAATTAAACTCTAGTATCTTATTAATTTC |
| 183 | GTATCTATGATAGTACCAATCAGGGAACATTAAACGGGTAAAGGTAAAG |
| 184 | CCCTGATTGGTACTATCATAGATACTACAAATTAAACTCTAGTATC |
| 185 | CCAGGGTATGATAGTACCAATAACATTAAACGGGTAAAGGTAAAGGAC |
| 186 | GTTATTGGTACTATCATACCCTGGATACTACAAATTAAACTCTAGTATC |
| 187 | GGTAAAGGACAAATATGATAGTACCAATCGAACATGGCGATTAAAGGCGC |
| 188 | GTTCGATTGGTACTATCATATTTGTCCTTTACCTTTACCCGTTTAATG |
| 190 | CTTATGATAGTACCAATAAGCTTGTTTAAATTGAAACTTCGTTATATTC |
| 191 | CTTATTGGTACTATCATAAGTAATAAAGAAACTCAACGGTTTATTAAGAC |
| 200 | AGCGACGCCGCAGGGGGATCCTGATGTAACGCTTATATTATTTTAAG |
| 201 | TGATATCTCCTTCTTAAAGTTCATGGGTCGTGGCTGTCTGTGGTGTC |
| 203 | AGCGACGCCGCAGGGGGATCCGTATCAATGGAATCCGGGACGC |
| 204 | TGATATCTCCTTCTTAAAGTTCAGACAATATGTAAGCCTTCGGCTGC |
| 211 | GATACTAGAGTTTATGATAGTACCAATTAATTTGTAGTATCCAGGGAAC |
| 212 | AATTAATTGGTACTATCATAAACTCTAGTATCTTATTAATTTCTGTTAC |
| 243 | GATACTATATGATAGTACCAATGAGTTTAATTTGTAGTATCCAGGG |
| 244 | CTCATTGGTACTATCATATAGTATCTTATTAATTTCTGTTACTATTAAC |
| 245 | GATACTAGTATGATAGTACCAATAGTTTAATTTGTAGTATCCAGGGAAC |
| 246 | CTATTGGTACTATCATACTAGTATCTTATTAATTTCTGTTAC |
| 247 | GATACTAGATATGATAGTACCAATGTTTAATTTGTAGTATCCAGGGAAC |
| 248 | CATTGGTACTATCATATCTAGTATCTTATTAATTTCTGTTAC |
| 249 | GTATGATAGTACCAATTTTAATTTGTAGTATCCAGGGAACATTAAAC |
| 250 | AATTAAAATTGGTACTATCATACTCTAGTATCTTATTAATTTCTGTTAC |
| 251 | GAGTTTTATGATAGTACCAATAATTTGTAGTATCCAGGGAACATTAAAC |
| 252 | AATTATTGGTACTATCATAAAACTCTAGTATCTTATTAATTTCTGTTAC |
| 253 | GAGTTTATATGATAGTACCAATATTTGTAGTATCCAGGGAACATTAAAC |
| 254 | CTACAAATATTGGTACTATCATATAAACTCTAGTATCTTATTAATTTCTG |
| 255 | GAGTTTAATATGATAGTACCAATTTTGTAGTATCCAGGGAACATTAAACG |
| 256 | CTACAAAATTGGTACTATCATATTAAACTCTAGTATCTTATTAATTTCTG |
| 259 | TGAACTTTAAGAAGGAGATATCATATGGATAGCACTGAGAACGTCATC |
| 260 | AAAACGACGGCCAGTACTAGCTACTGGAACAGGTGGTGG |
| 261 | GAAAGGCTCAGTCGAAAGACTGGGCCTTTCGTTTTATCTACTGGAACAGGTGGTGG |
| 262 | AAAACGACGGCCAGTACTAGGTATCAATGGAATCCGGGACGC |
| 263 | GTCTTTCGACTGAGCCTTTCGTTTTATTTTACTTGTACAGCTCGTCCATG |
| 379 | GATACTAGAGTCATTAATGATAATGCTTAATTTGTAGTATCCAGGGAAC |
| 380 | AAATTAAGCATTATCATTAATGACTCTAGTATCTTATTAATTTCTGTTAC |
| 381 | GATACTAGAGTTATGATAGTAGCAATTTAATTTGTAGTATCCAGGGAAC |
| 382 | AAATTAAATTGCTACTATCATAACTCTAGTATCTTATTAATTTCTGTTAC |
| 385 | AGCGACGCCGCAGGGGGATCCGTGTAGTGATTTCTGTTGCAGGTTTATG |
| 386 | AGCGACGCCGCAGGGGGATCCATATGTTAATAGTAACAGAAATTAATAAG |
| 387 | AGCGACGCCGCAGGGGGATCCCACTCTCGCAACACTCGCTC |
| 388 | TGATATCTCCTTCTTAAAGTTCAGATCTTGTGCGGTCTAGATAATGCG |
| 389 | TATGATAGTACCAATAATAACTAGTATTTTTAATGACTTAC |
| 390 | CTAGTTATTATTGGTACTATCATATATTTACTAAATATAGAAGTTAATTG |
| 393 | CACAATTATGATAGTACCAATTATTTCATACATGTGTTATGTTAATTAC |
| 394 | ATTGGTACTATCATAATTGTGAATACTAAAGTTAATAGTTTC |
| 395 | AGCGACGCCGCAGGGGGATCCATTATGTGCGTATCGCTGCTGCTC |
| 396 | TGATATCTCCTTCTTAAAGTTCATCTGCGCTCGCCAGCAAGTG |
| 397 | GTTAGTTATGATAGTACCAATATATTAATTACTTGATTTAATTGAAGGGG |
| 398 | ATTGGTACTATCATAACTAACTTAATTGAGTTTATTTTTACGG |
| 404 | AAAACGACGGCCAGTACTAGGTCACGACCTGATTTTTTACCTCG |
| 408 | AGCGACGCCGCAGGGGGATCCCGATGTATGTCAGCAATGAGGTTG |
| 409 | TGATATCTCCTTCTTAAAGTTCAGTCATCCGACCCGCCATTATTC |
| 410 | GAAACTTTATGATAGTACCAATCAATAACTTAGTAACTTTAAAAGGGG |
| 411 | GATTGGTACTATCATAAAGTTTCTAGAATATAAGTTATTACACAAAAACG |
| 412 | AGCGACGCCGCAGGGGGATCCATTTGGAATCTTGGTACTTTGGTGGC |
| 413 | TGATATCTCCTTCTTAAAGTTCAAGGTTGCGGTTCTAAAACTGTCG |
| 414 | CAATTTTAATGTGTATGATAGTACCAATTTAAAGTTTCTATATTCCATTCTAAAATAAC |
| 415 | AATTGGTACTATCATACACATTAAAATTGAATATTAAATGTTGTTTAAAG |
| 416 | AGCGACGCCGCAGGGGGATCCGCAGATTCAGTGGTTCACCAC |
| 417 | TGATATCTCCTTCTTAAAGTTCAGCTTTTCTCTGCACAGTATTTGTCAT |
| 418 | TAAGTTATGATAGTACCAATTTGTCATTAAAGAAATGGGGTAGAGCTATG |
| 419 | CAAATTGGTACTATCATAACTTATTTTAACTATTTAAAGACTAATTAAATTTAAC |
| 435 | AGCGACGCCGCAGGGGGATCCTTTCCTGCCTTGACATGCGAGAAC |
| 436 | TGATATCTCCTTCTTAAAGTTCAGACTGTGGAGATGATGCGGAATTTC |
| 437 | TAAAATATCGTATGATAGTACCAATGAGTTTATTTATGTGATTTGACCGG |
| 438 | CATTGGTACTATCATACGATATTTTAATCAGATAAATAATTACTAATAAC |
| 439 | AGCGACGCCGCAGGGGGATCCATCCCGTACAGGTATTTTGCGTAGTG |
| 440 | TGATATCTCCTTCTTAAAGTTCAATCTTTATCCTTGCTTCGAAAATTAGC |
| 441 | CAAGTATGATAGTACCAATGTGATTATGCTTCACATTAATACTTAATAAG |
| 442 | CATAATCACATTGGTACTATCATACTTGTCAAACAAATGCAATACTTTTC |
| 444 | AGCGACGCCGCAGGGGGATCCCATATACCCAAGCACTTGGCGATC |
| 445 | TGATATCTCCTTCTTAAAGTTCAAGTTGATGTGGCTGACGTGGTG |
| 446 | CGCGTTTATGATAGTACCAATTCTCTATTTTAATTAATATAATTAATGTAGTTTATTAAC |
| 447 | ATTAAAATAGAGAATTGGTACTATCATAAACGCGATAGGCGTGTATGTGG |
| 449 | AGCGACGCCGCAGGGGGATCCTGCTGCACGAATGCGTAACCTC |
| 450 | TGATATCTCCTTCTTAAAGTTCAGCCGAATATTCGGCGAGTTTTTAG |
| 451 | GAAAAGTCACTTATGATAGTACCAATTAAGCGCACGCTAAAAGCGAATTG |
| 452 | GCGCTTAATTGGTACTATCATAAGTGACTTTTCAATTGTGCTGTAATTG |
| 459 | AGCGACGCCGCAGGGGGATCCTGGAGTCGCGGGTGCTCAAC |
| 460 | TGATATCTCCTTCTTAAAGTTCATACCTCTCGACCCTGTTTATTAAAAG |
| 497 | AGCGACGCCGCAGGGGGATCCGCGCCTCTACGAGACATTGG |
| 498 | GATATCTCCTTCTTAAAGTTCAATAAGGCGAAATACATTTAATAGCAATC |
| 501 | AGCGACGCCGCAGGGGGATCCTATCAGGACGGCTCCTATATCGG |
| 502 | GATTTATAACTTCCCCAAATATCAATGTGCGTTTGTGAACGACCGCTC |
| 503 | GCGGTCGTTCACAAACGCACATTGATATTTGGGGAAGTTATAAATCAAG |
| 504 | AAAACGACGGCCAGTACTAGTGCACGGGCTAGACGACCTAG |
| 505 | TGATATCTCCTTCTTAAAGTTCAAGACGCGAGCGGTCGTTCAC |
| 516 | AGCGACGCCGCAGGGGGATCCGCCTCCTCATCGATTTCCGC |
| 517 | AAAACGACGGCCAGTACTAGCCGTAGCAGCGCTTGTCTCG |
| 520 | AGCGACGCCGCAGGGGGATCCGACCACTACCTCAAGGGCGG |
| 521 | AAAACGACGGCCAGTACTAGCAGTTGCAGACGTGTACGACAC |
| 560 | AGCGACGCCGCAGGGGGATC |
| 570 | GTATATTTATGATAGTACCAATAATTACTTGATTTAATTGAAGGGGTTTA |
| 571 | TTATTGGTACTATCATAAATATACTAACTTAATTGAGTTTATTTTTACGG |
| 572 | AGTATGATAGTACCAATTTAGTATATTAATTACTTGATTTAATTGAAGGG |
| 573 | TACTAAATTGGTACTATCATACTTAATTGAGTTTATTTTTACGGTTTAAT |
| 574 | AAATTATATGATAGTACCAATAACCGTAAAAATAAACTCAATTAAGTTAG |
| 575 | TTTTACGGTTATTGGTACTATCATATAATTTACTCTAAGCGAAGACGCCC |
| 576 | TATGATAGTACCAATATAAACTCAATTAAGTTAGTATATTAATTACTTGATTTAATTG |
| 577 | TGAGTTTATATTGGTACTATCATATTTTACGGTTTAATTTACTCTAAGCG |
| 578 | AATATGATAGTACCAATTTAAGTTAGTATATTAATTACTTGATTTAATTG |
| 579 | CTTAAATTGGTACTATCATATTGAGTTTATTTTTACGGTTTAATTTACTC |
| 580 | TATTAATTATATGATAGTACCAATCTTGATTTAATTGAAGGGGTTTATAG |
| 581 | TTGGTACTATCATATAATTAATATACTAACTTAATTGAGTTTATTTTTAC |
| 601 | ATGATATCTCCTTCTTAAAGTTCATAAAAAGCCGTAACG |
| 617 | CAGAAATTAATAAGATACTTCTCAAATTAAACATCATAGGTCCCTTG |
| 618 | TAATTTGAGAAGTATCTTATTAATTTCTGTTACTATTAACATATAGGTTT |
| 619 | ACAGAAATTAATAAGATACTAGAGTTTAATTTGTAGTATCGTCCCTTGTAATTTGCCCATTTCC |
| 620 | AAAACGACGGCCAGTACTAGTTACTTGTACAGCTCGTCCATGCC |
| E115 | TTATTCGAAAGGAATGCCTTCTTTTTCG |
| E227 | GACCATGATTACGCCAAGCTTGAAAATCGGGAGTGGGAAAGAG |
| E228 | GTAGCTACACAGACGACCAGATTCGTGGGCGAAGTGGTTC |
| E229 | AAGGCATTCCTTTCGAATAAGTGAACTCAGAAATGCCAGGATTTG |
| E230 | AAAACGACGGCCAGTGAATTCCAAGACCGTCACCCAACTAG |
| E245 | CTGGTCGTCTGTGTAGCTAC |

**Table S2E: Oligonucleotides used for the amplification of DNA probes for surface plasmon resonance analysis.** Primers belonging to the same first PCR reaction are alternately shadowed in grey or white. The first PCR product was used as template for the second PCR reaction. The first primer was combined with the primer Biotin-primer (Biotin-GAGGAGTCGTCGATGTGGAGACC), which was 5´-fused to biotin.

| Sample | Oligo-nucleotide number | Sequence (5´🡪3´) | Template |
| --- | --- | --- | --- |
| P_cg3336_ (control) | M259 | CGCCCTCACCGGTGG | pJC1-P_cg3336_ |
|  | M260 | GAGGAGTCGTCGATGTGGAGACCCCAAGATCTTTGGGGAGAGATTTTT |  |
| P_cg1999_ | 710 | ACGTGCCCGATCTGCTGGATC | pJC1-P_cg1999_ |
|  | 709 | GAGGAGTCGTCGATGTGGAGACCATTTGTGGGCGCTTTAATCGCTAAC |  |
| P_cg1999__A-T/G-C | 507 | TGCACGGGCTAGACGACCTAG | pJC1-P_cg1999__A-T/G-C |
|  | 506 | GAGGAGTCGTCGATGTGGAGACCTAAACACCCGCGAAATTAGCGATTG |  |
| P*_lys_* | M263 | CTCGACGACGGCCACTG | *C. glutamicum* genome |
|  | M264 | GAGGAGTCGTCGATGTGGAGACCAGTGCCTTCTTTGAGGCTTGA |  |
| P*_lys_*_CS_0 | M263 | CTCGACGACGGCCACTG | pJC1-P*_lys_*_CS_0 |
|  | M264 | GAGGAGTCGTCGATGTGGAGACCAGTGCCTTCTTTGAGGCTTGA |  |

**Table S2F: Oligonucleotides used for electrophoretic mobility shift assays.** Primers and their corresponding templates are alternately shadowed in grey or white.

| Sample | Oligo-nucleotide number | Sequence (5´🡪3´) | Template |
| --- | --- | --- | --- |
| P*_lys_* | 117 | AGCGACGCCGCAGGGGGATCCGCTCAAGGAAGAGTTCTTCATTGGTC | pJC1-P*_lys_*-*venus* |
|  | 114 | TGATATCTCCTTCTTAAAGTTCAATTTTTCGGCATTGCGCCTTTAATCGC |  |
| P*_lys_*_CS_0 | 117 | AGCGACGCCGCAGGGGGATCCGCTCAAGGAAGAGTTCTTCATTGGTC | pJC1-P*_lys_*_CS_0-*venus* |
|  | 114 | TGATATCTCCTTCTTAAAGTTCAATTTTTCGGCATTGCGCCTTTAATCGC |  |

**Table S2G: Oligonucleotides used for sequencing.**

| Oligonucleotide name | Sequence (5´🡪3´) | Target |
| --- | --- | --- |
| pJC1-MCS-fw | CAGGGACAAGCCACCCGCACA | All pJC1-based plasmids (Table S2C) |
| pJC1-MCS-rv | GGAAGCTAGAGTAAGTAGTTCGC |  |
| R274-eYFPK5-seq-rv | GCAGGACCATGTGATCGCGC | pJC1-P*_lys_*_CS_0-*venus*-T-P*_gntK_*-*e2-crimson* |
| R216-venus-end-fw | CTCTCGGCATGGACGAGCTGTAC |  |
| 492-venus-seq-rv | CTCGAACTTCACCTCGGCGC |  |
| M13-fw | CGCCAGGGTTTTCCCAGTCAC | pK19*mobsacB*-1199_1201-*cgpS* |
| M13-rv | AGCGGATAACAATTTCACACAGGA |  |
| cg1199_1201_seq_fw | GTGGAAAAAATTGGGGTTTCCG | Genomically re-integrated P*_cgpS_*-*cgpS* sequence in the intergenic region of cg1199-cg1201 |
| cg1199_1201_seq_rv | GCTCATTGTCACAGATGGCG |  |

**Table S2H: Ordered DNA sequences.** All DNA sequences were synthesized by Eurofins Genomics (Ebersberg, Germany) and provided as plasmids or DNA strands.

| Ordered Sequences  Plasmids | Sequence (5´🡪3´) |
| --- | --- |
| pEX-K168-P_cg1999__A-T/G-C | TATCAGGACGGCTCCTATATCGGTGGGCAGGACAGCTCTCGCGTTGTTGTTCACGAGAACGACCTAAACGACAACCAATATAAACACCCGCGAAATTAGCGATTGTGAATTTTGTGAAGAGTTGCGAAGATAGACAGTCGACACATCTACCAGCACCGCCTTAGTAACAGTCGTATCGATATAAAATGTTATACGAGTTAATCAAAGACGCAGACCTTTGCCACGAATACCACTAAGGCCCGCAGAAGCGAATCTCATTTAAAAACCGTAAAAATAAACTCAATTAAGTTAGTATATTAATTACTTGATTTATAACTTCCCCAAATATCATACTGCGTTTGTGAACGACCGCTCGCGTCTTAGCAGTGGTGCCGTCTCGAATTTCGCTACGACCGTGCTCCCCTATTCCACAAAGACGGACCACAGCGTCGCGCCCGCGGTCTCTAGGAGCTGTTGAAGCTAGGTCGTCTAGCCCGTGCA |
| pEX-K168- P_cg1999__rand | GCGCCTCTACGAGACATTGGATGACCTAGGCTAACTAGGCTTATAGAAGAGGGCACGCACGTACGGTATCGGAGCTTTCATTGCCATTGGTAGGATCAAAACCCGTGACGAGTTGCTCTCCCATGCTACTATCAGAACTGACAATTTGTAGAGGCCCTGAACGCTTTCAGCCGATTACACACAAGTGGTGGACTAATATTGGGGAGGCTGCTGCCCAGATCAATGAGCCTTAGGAGCAGATGATTAATACCGGCGCAATTAAACCGTAAAAATAAACTCAATTAAGTTAGTATATTAATTACTTGATTTACCTTACCGGATCACGTTAATGATTGCTATTAAATGTATTTCGCCTTATATCTGCCTCGCTCAAGGTTGCTTAAGCTGGCAACTGTTATTATATGGGTTGCCCGAGCACGGATGGCTCGCTTCAACTCCGCATTGACAGTTTACACACCCGCCGAGACGGGGATTCCGACT |
| pEK-A2-P*_lys_*::GntR_BS_pos-50 | GCTCAAGGAAGAGTTCTTCATTGGTCTTTTGTTGCGGGTTGCCGTAGATGATTTCAGGGTGGCTCAAAGTCAGGGAGGTGTAGTCGAATTCGATTGGGTGAAGCCTGTCGCGATTACGGTTTTCGCGCTCGGTACCAGTAATAAAGCTTGCGATGCGGGAGTAATTAACCCGACCTTTGATAAATACCTGAGTGCCTTCTTTGAGGCTTGATGCCTTGATCTTTTCGGCGACTGGTGCTGGCTGCACAGGTTGAGGGGTAACTGGGTAGGTCATGAGAGAAAAAGCCTTTCGAGAACTGGGTGTAGTGATTTCTGTTGCAGGTTTATGTAGACGAAACTTATGATAGTACCAATTTAAAAAACCTATATGTTAATAGTAACAGAAATTAATAAGATACTAGAGTTTAATTTGTAGTATCCAGGGAACATTAAACGGGTAAAGGTAAAGGACAAACGAACATGGCGATTAAAGGCGCAATGCCGAAAAAT |
| Ordered Sequences  Gene strands |  |
| P*_lys_*_A-T/G-C | AGCGACGCCGCAGGGGGATCCCGAGTTCCTTCTCAAGAAGTAACCAGAAAACAACGCCCAACGGCATCTACTAAAGTCCCACCGAGTTTCAGTCCCTCCACATCAGCTTAAGCTAACCCACTTCGGACAGCGCTAATGCCAAAAGCGCGAGCCATGGTCATTATTTCGAACGCTACGCCCTCATTAATTGGGCTGGAAACTATTTATGGACTCACGGAAGAAACTCCGAACTACGGAACTAGAAAAGCCGCTGACCACGACCGACGTGTCCAACTCCCCATTGACCCATCCAGTACTCTCTTTTTCGGAAAGCTCTTGACCCACAAGTGATTTCTGTTGCAGGTTTATGTAGACGAAACTTTAAAAAACCTATATGTTAATAGTAACAGAAATTATATTCTATGATCTCAAATTAAACATCATAGGTCCCTTGTAATTTGCCCATTTCCATTTCCTGTTTGCTTGATGCGCTAATTTCCGCGTTACGGCTTTTTATGAACTTTAAGAAGGAGATATCAT |

**Table S2I: Sequences of the native phage promoters P_cg1999_ and P*_lys_* and the corresponding synthetic variants shown in Figure 2.** Highlighted are the core promoter region (blue), the ATG as translational start codon (bold red) and the stop codon introduced after 30 nucleotides (bold yellow). For the native P*_lys_* promoter, the 100 bp core promoter region is highlighted.

| Promoter | Sequence (5´🡪3´) |
| --- | --- |
| P_cg1999_ | |
| P_cg1999_ | ATAGTCCTGCCGAGGATATAGCCACCCGTCCTGTCGAGAGCGCAACAACAAGTGCTCTTGCTGGATTTGCTGTTGGTTATATTTGTGGGCGCTTTAATCGCTAACACTTAAAACACTTCTCAACGCTTCTATCTGTCAGCTGTGTAGATGGTCGTGGCGGAATCATTGTCAGCATAGCTATATTTTACAATATGCTCAATTAGTTTCTGCGTCTGGAAACGGTGCTTATGGTGATTCCGGGCGTCTTCGCTTAGAGTAAATTAAACCGTAAAAATAAACTCAATTAAGTTAGTATATTAATTACTTGATTTAATTGAAGGGGTTTATAGT**ATG**ACGCAAACACTTGCTGGCGAGCGCAGA**TGA** |
| P_cg1999__A-T/G-C | TATCAGGACGGCTCCTATATCGGTGGGCAGGACAGCTCTCGCGTTGTTGTTCACGAGAACGACCTAAACGACAACCAATATAAACACCCGCGAAATTAGCGATTGTGAATTTTGTGAAGAGTTGCGAAGATAGACAGTCGACACATCTACCAGCACCGCCTTAGTAACAGTCGTATCGATATAAAATGTTATACGAGTTAATCAAAGACGCAGACCTTTGCCACGAATACCACTAAGGCCCGCAGAAGCGAATCTCATTTAAAAACCGTAAAAATAAACTCAATTAAGTTAGTATATTAATTACTTGATTTATAACTTCCCCAAATATCA**ATG**TGCGTTTGTGAACGACCGCTCGCGTCT**TGA** |
| P_cg1999__rand | GCGCCTCTACGAGACATTGGATGACCTAGGCTAACTAGGCTTATAGAAGAGGGCACGCACGTACGGTATCGGAGCTTTCATTGCCATTGGTAGGATCAAAACCCGTGACGAGTTGCTCTCCCATGCTACTATCAGAACTGACAATTTGTAGAGGCCCTGAACGCTTTCAGCCGATTACACACAAGTGGTGGACTAATATTGGGGAGGCTGCTGCCCAGATCAATGAGCCTTAGGAGCAGATGATTAATACCGGCGCAATTAAACCGTAAAAATAAACTCAATTAAGTTAGTATATTAATTACTTGATTTACCTTACCGGATCACGTTA**ATG**ATTGCTATTAAATGTATTTCGCCTTAT**TGA** |
| P*_lys_* | |
| P*_lys_* | GCTCAAGGAAGAGTTCTTCATTGGTCTTTTGTTGCGGGTTGCCGTAGATGATTTCAGGGTGGCTCAAAGTCAGGGAGGTGTAGTCGAATTCGATTGGGTGAAGCCTGTCGCGATTACGGTTTTCGCGCTCGGTACCAGTAATAAAGCTTGCGATGCGGGAGTAATTAACCCGACCTTTGATAAATACCTGAGTGCCTTCTTTGAGGCTTGATGCCTTGATCTTTTCGGCGACTGGTGCTGGCTGCACAGGTTGAGGGGTAACTGGGTAGGTCATGAGAGAAAAAGCCTTTCGAGAACTGGGTGTAGTGATTTCTGTTGCAGGTTTATGTAGACGAAACTTTAAAAAACCTATATGTTAATAGTAACAGAAATTAATAAGATACTAGAGTTTAATTTGTAGTATCCAGGGAACATTAAACGGGTAAAGGTAAAGGACAAACGAAC**ATG**GCGATTAAAGGCGCAATGCCGAAAAAT**TGA** |
| P*_lys_*_A-T/G-C_70 bp | CGAGTTCCTTCTCAAGAAGTAACCAGAAAACAACGCCCAACGGCATCTACTAAAGTCCCACCGAGTTTCAGTCCCTCCACATCAGCTTAAGCTAACCCACTTCGGACAGCGCTAATGCCAAAAGCGCGAGCCATGGTCATTATTTCGAACGCTACGCCCTCATTAATTGGGCTGGAAACTATTTATGGACTCACGGAAGAAACTCCGAACTACGGAACTAGAAAAGCCGCTGACCACGACCGACGTGTCCAACTCCCCATTGACCCATCCAGTACTCTCTTTTTCGGAAAGCTCTTGACCCACAAGTGATTTCTGTTGCAGGTTTATGTAGACGAAACTTTAAAAAACCTATATGTTAATAGTAACAGAAATTATATTCTATGATCTCAAATTAAACATCATAGGTCCCTTGTAATTTGCCCATTTCCATTTCCTGTTTGCTTG**ATG**CGCTAATTTCCGCGTTACGGCTTTTTA**TGA** |
| P*_lys_*_A-T/G-C_80 bp | CGAGTTCCTTCTCAAGAAGTAACCAGAAAACAACGCCCAACGGCATCTACTAAAGTCCCACCGAGTTTCAGTCCCTCCACATCAGCTTAAGCTAACCCACTTCGGACAGCGCTAATGCCAAAAGCGCGAGCCATGGTCATTATTTCGAACGCTACGCCCTCATTAATTGGGCTGGAAACTATTTATGGACTCACGGAAGAAACTCCGAACTACGGAACTAGAAAAGCCGCTGACCACGACCGACGTGTCCAACTCCCCATTGACCCATCCAGTACTCTCTTTTTCGGAAAGCTCTTGACCCACAAGTGATTTCTGTTGCAGGTTTATGTAGACGAAACTTTAAAAAACCTATATGTTAATAGTAACAGAAATTAATAAGATACTTCTCAAATTAAACATCATAGGTCCCTTGTAATTTGCCCATTTCCATTTCCTGTTTGCTTG**ATG**CGCTAATTTCCGCGTTACGGCTTTTTA**TGA** |
| P*_lys_*_A-T/G-C_100 bp | CGAGTTCCTTCTCAAGAAGTAACCAGAAAACAACGCCCAACGGCATCTACTAAAGTCCCACCGAGTTTCAGTCCCTCCACATCAGCTTAAGCTAACCCACTTCGGACAGCGCTAATGCCAAAAGCGCGAGCCATGGTCATTATTTCGAACGCTACGCCCTCATTAATTGGGCTGGAAACTATTTATGGACTCACGGAAGAAACTCCGAACTACGGAACTAGAAAAGCCGCTGACCACGACCGACGTGTCCAACTCCCCATTGACCCATCCAGTACTCTCTTTTTCGGAAAGCTCTTGACCCACAAGTGATTTCTGTTGCAGGTTTATGTAGACGAAACTTTAAAAAACCTATATGTTAATAGTAACAGAAATTAATAAGATACTAGAGTTTAATTTGTAGTATCGTCCCTTGTAATTTGCCCATTTCCATTTCCTGTTTGCTTG**ATG**CGCTAATTTCCGCGTTACGGCTTTTTA**TGA** |

**REFERENCES**

1. Studier FW, Moffatt BA. 1986. Use of bacteriophage T7 RNA polymerase to direct selective high-level expression of cloned genes. J Mol Biol 189:113-130.

2. Kinoshita S, Udaka S, Shimono M. 1957. Studies on the amino acid fermentation. Part 1. Production of L-glutamic acid by various microorganisms. J Gen Appl Microbiol 3:193-205.

3. Frunzke J, Engels V, Hasenbein S, Gätgens C, Bott M. 2008. Co-ordinated regulation of gluconate catabolism and glucose uptake in *Corynebacterium glutamicum* by two functionally equivalent transcriptional regulators, GntR1 and GntR2. Mol Microbiol 67:305-322.

4. Baumgart M, Unthan S, Rückert C, Sivalingam J, Grünberger A, Kalinowski J, Bott M, Noack S, Frunzke J. 2013. Construction of a prophage-free variant of *Corynebacterium glutamicum* ATCC 13032 for use as a platform strain for basic research and industrial biotechnology. Appl Environ Microbiol 79:6006-6015.

5. Cremer J, Eggeling L, Sahm H. 1990. Cloning the *dapA* *dapB* cluster of the lysine-secreting bacterium *Corynebacterium glutamicum*. Mol Gen Genet 220:478-480.

6. Nanda AM, Heyer A, Krämer C, Grünberger A, Kohlheyer D, Frunzke J. 2014. Analysis of SOS-induced spontaneous prophage induction in *Corynebacterium glutamicum* at the single-cell level. J Bacteriol 196:180-188.

7. Schäfer A, Tauch A, Jäger W, Kalinowski J, Thierbach G, Pühler A. 1994. Small mobilizable multi-purpose cloning vectors derived from the *Escherichia coli* plasmids pK18 and pK19: selection of defined deletions in the chromosome of *Corynebacterium glutamicum*. Gene 145:69-73.

8. Pfeifer E, Hünnefeld M, Popa O, Polen T, Kohlheyer D, Baumgart M, Frunzke J. 2016. Silencing of cryptic prophages in *Corynebacterium glutamicum*. Nucleic Acids Res 44:10117-10131.
