# Supplementary figures and images for: Deciphering the Rules Underlying Xenogeneic Silencing and Counter-Silencing of Lsr2-like Proteins Using CgpS of Corynebacterium glutamicum as a Model
Source: mBio. 2020 Feb 4;11(1):e02273-19. doi: 10.1128/mBio.02273-19 (PMC7002338; doi:10.1128/mBio.02273-19)

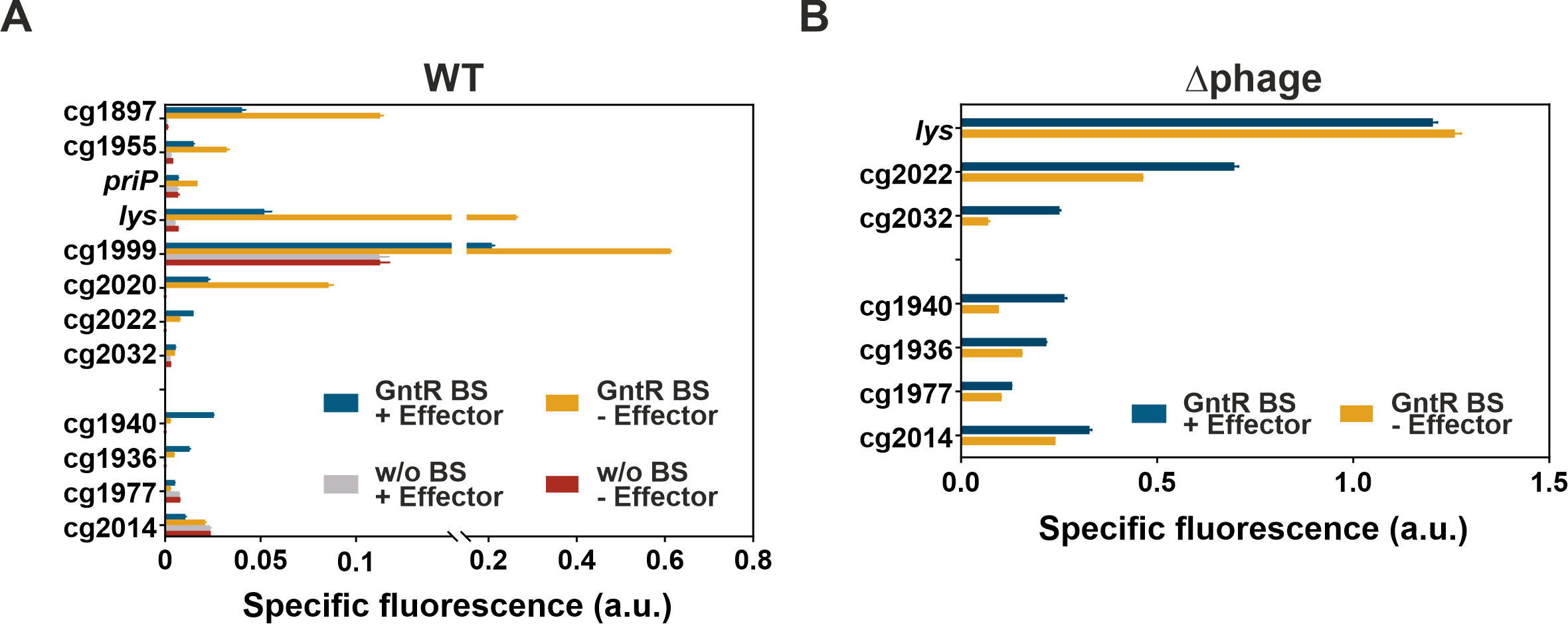

Supplement: FIG S1 [file mBio.02273-19-sf001.tif]

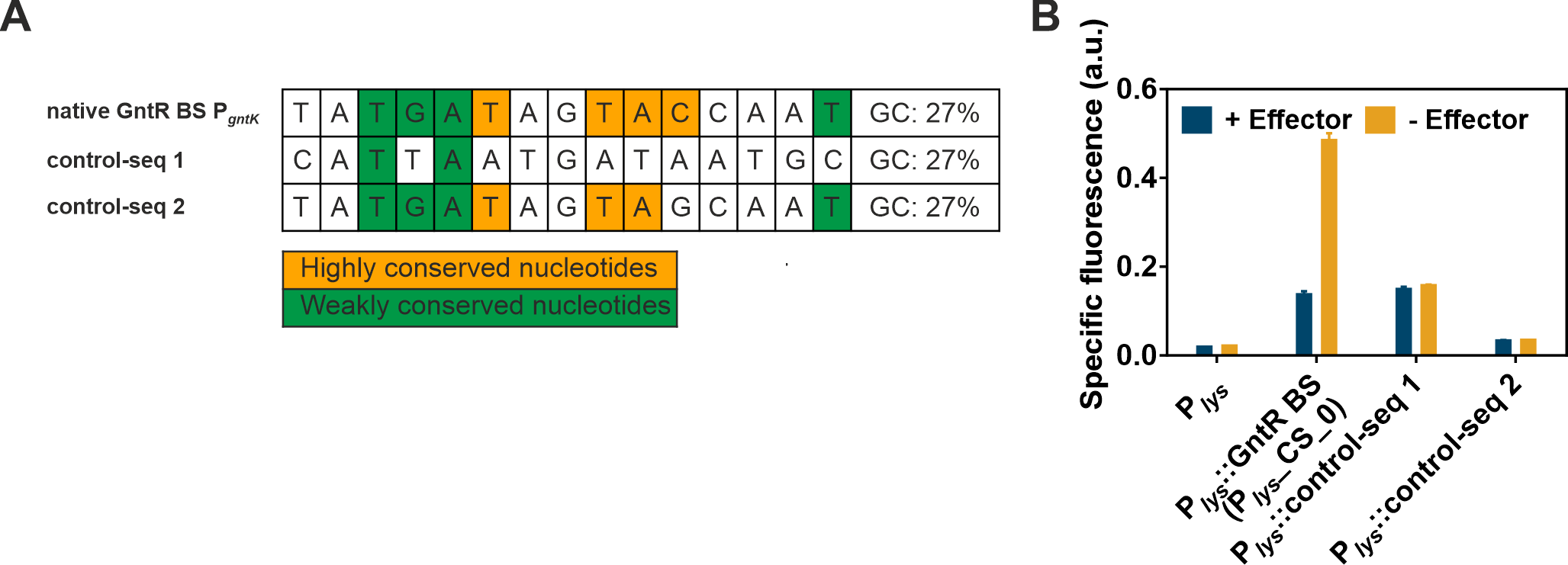

Supplement: FIG S2 [file mBio.02273-19-sf002.tif]

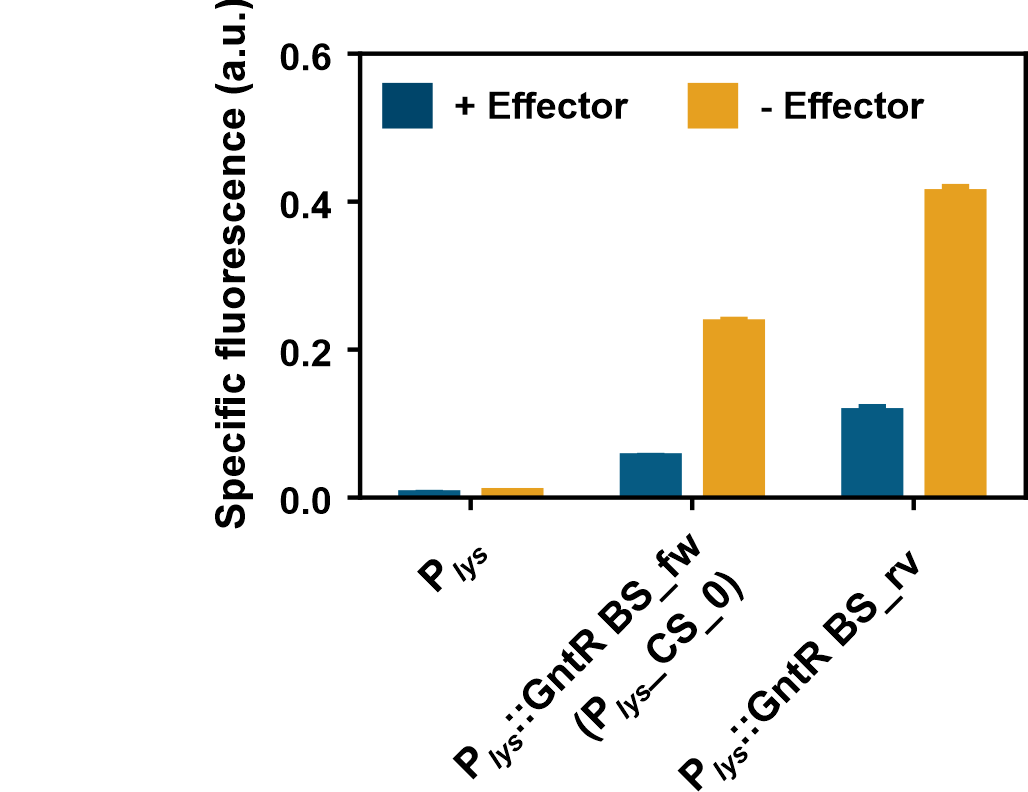

Supplement: FIG S3 [file mBio.02273-19-sf003.tif]

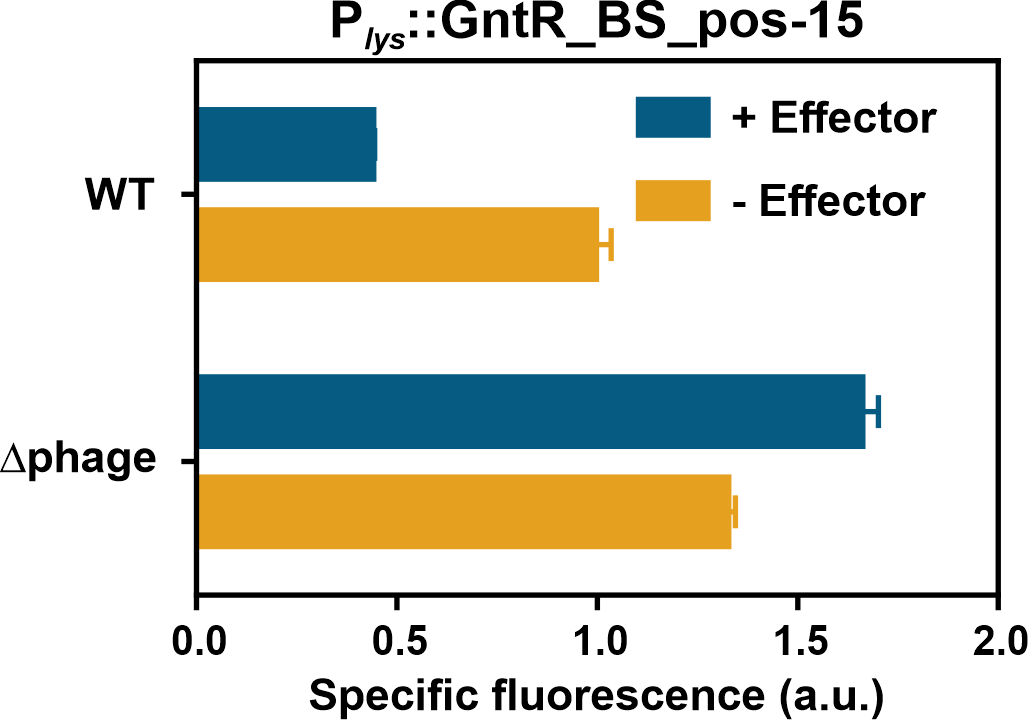

Supplement: FIG S4 [file mBio.02273-19-sf004.tif]

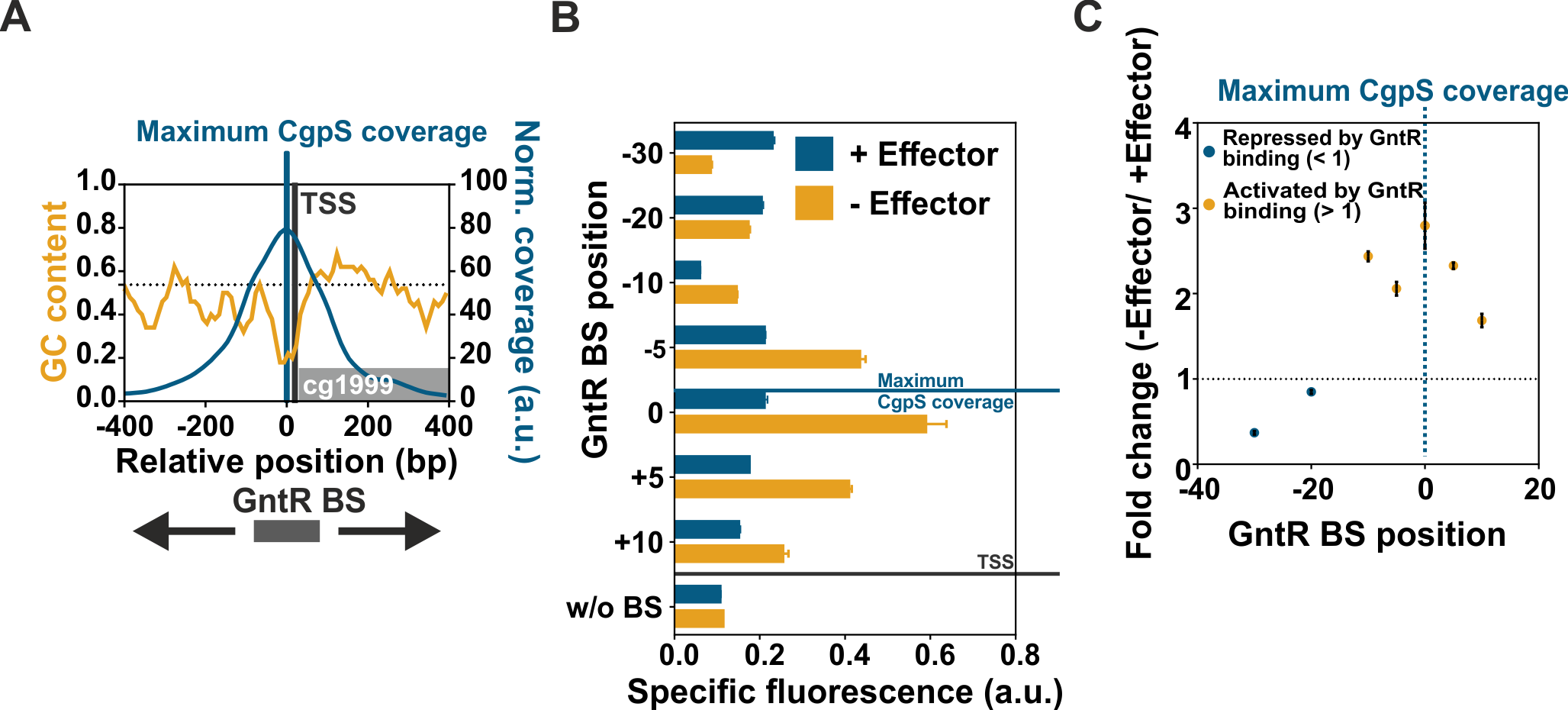

Supplement: FIG S5 [file mBio.02273-19-sf005.tif]

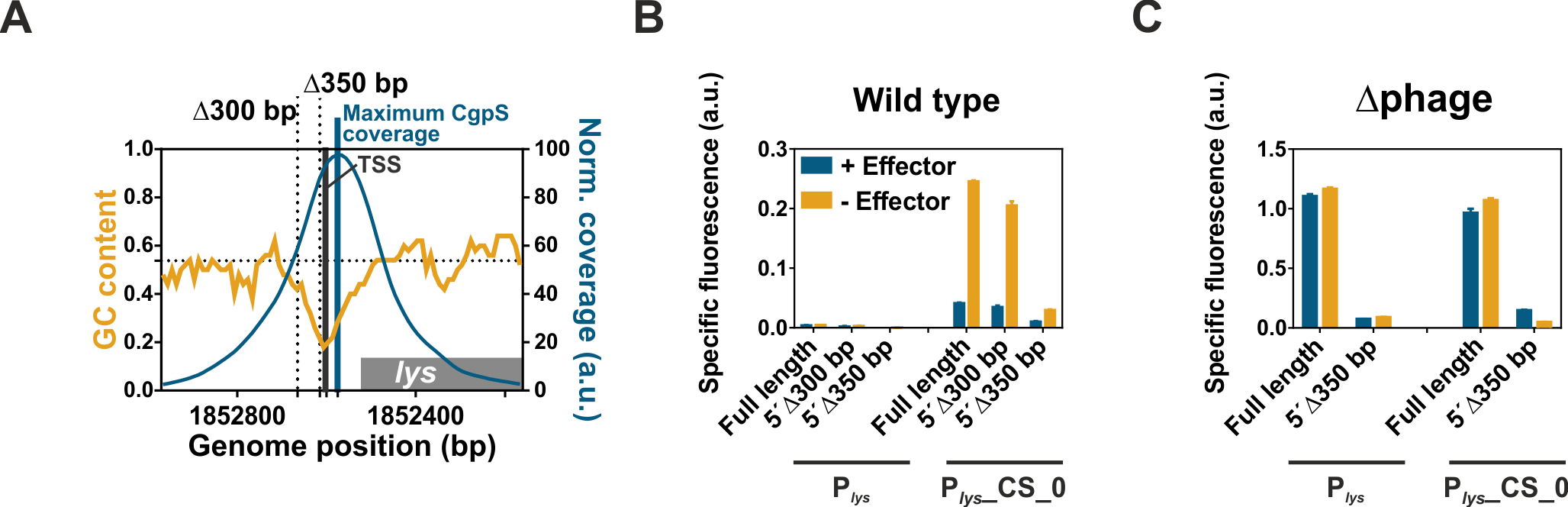

Supplement: FIG S6 [file mBio.02273-19-sf006.tif]
